# Supplementary material for: OFD1 inhibition induces BRCAness to create a therapeutic vulnerability to PARP inhibition in pancreatic cancer
Source: Nat Commun. 2025 Aug 5;16:7209. doi: 10.1038/s41467-025-62295-8 (PMC12325586; doi:10.1038/s41467-025-62295-8)

Supplementary information

OFD1 inhibition induces BRCAness to create a therapeutic vulnerability to PARP inhibition in pancreatic cancer

Peng Li[1+], Junjie Ye[1+], Qian Yang[1+], Ni Wang[1], Chaoyi Li[1], Xiaoxiao Zou[1], Hanyan Luo[1], Yi Pan[1], Lingxi Jian g[2,3,4], Baiyong Shen[2,3,4*], Zaiming Tang[1*], and Qing Zhong[1*]

[1]Institute for Translational Medicine on Cell Fate and Disease, Shanghai Ninth People's Hospital, Key Laboratory of Cell Differentiation and Apoptosis of Chinese Ministry of Education, Department of Pathophysiology, Shanghai Jiao Tong University School of Medicine (SJTU-SM), Shanghai 200025, China.

[2] Department of General Surgery, Pancreatic Disease Center, Ruijin Hospital, Shanghai Jiao Tong University School of Medicine, Shanghai 200025, China.

[3] Research Institute of Pancreatic Diseases, Shanghai Key Laboratory of Pancreatic Neoplasms Translational Research, Shanghai Jiao Tong University School of Medicine, Shanghai 200025, China.

[4] State Key Laboratory of Systems Medicine for Cancer, Institute of Translational Medicine, Shanghai Jiao Tong University, Shanghai 200025, China.

[+]These authors contributed equally to this study.

*To whom correspondence should be addressed. E-mail: qingzhong@shsmu.edu.cn, zaimingtang2017@shsmu.edu.cn, shenby@shsmu.edu.cn.

1. Supplementary Figures

2. Supplementary Table 1. Materials used in this study

3. Supplementary Table 2. Small molecule synthetic lethality screen targeting OFD1 in MIA PaCa-2 cells

4.Supplementary Table 3. PDX patients information table

5.Supplementary Table 4. Oligonucleotide sequences used in this paper.

6.Gating strategy for HR reporter assay by FACS

7.Source Data of Supplementary Figures

Supplementary Fig. 1

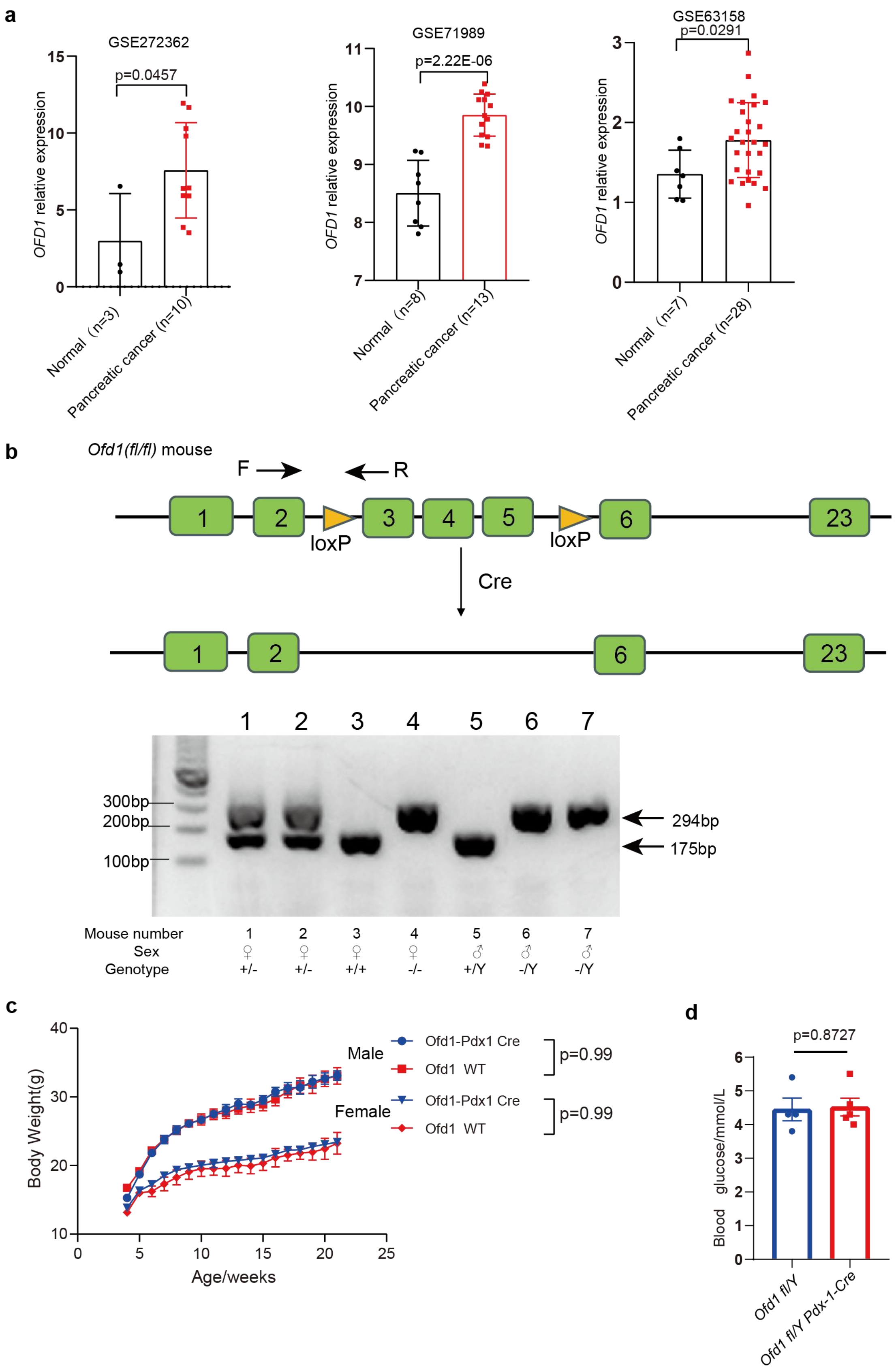

Supplementary Fig. 1 | Gene expression levels of *OFD1* in GEO pancreatic cancer datasets and characterization of pancreatic conditional knockout *Ofd1* mice.

a, *OFD1* Gene expression in tumor (T) versus normal (N) tissues. Data were obtained from publicly available GEO datasets (GSE272362, GSE71989, and GSE63158). Statistical analysis was performed using unpaired two-tailed Student's t-tests for each dataset. **b**, Generation and validation of pancreas-specific Ofd1 conditional knockout mice. Schematic representation of the strategy for generating the pancreatic conditional knockout *Ofd1* mice. Representative genotyping results confirm the *Ofd1* genotype in offspring from *Ofd1* conditional knockout and *Pdx1-Cre* mice after hybridization. **c**, Longitudinal analysis of body weight gain in *Ofd1*-wildtype and *Ofd1-Pdx1-Cre-KO* mice. Body weight gain of male and female *Ofd1-wildtype* (*wt*) and *Ofd1-Pdx1-Cre-KO* mice was recorded over a 21 weeks period. Statistical differences in body weight between the groups were assessed using two-way ANOVA followed by Tukey's post hoc test. Male (*Ofd1-wt* = 6 mice, *Ofd1-Pdx1-Cre-KO* = 6 mice); Female (*Ofd1-wt* = 3 mice, *Ofd1-Pdx1-Cre-KO* = 10 mice). **d**, Blood glucose levels in Ofd1-wt and Ofd1-Pdx1-Cre-KO mice. Fasting blood glucose was measured and compared between groups (*Ofd1-wt* = 4 mice; *KO* = 5 mice) using unpaired two-tailed Student's t-tests.

Supplementary Fig. 2

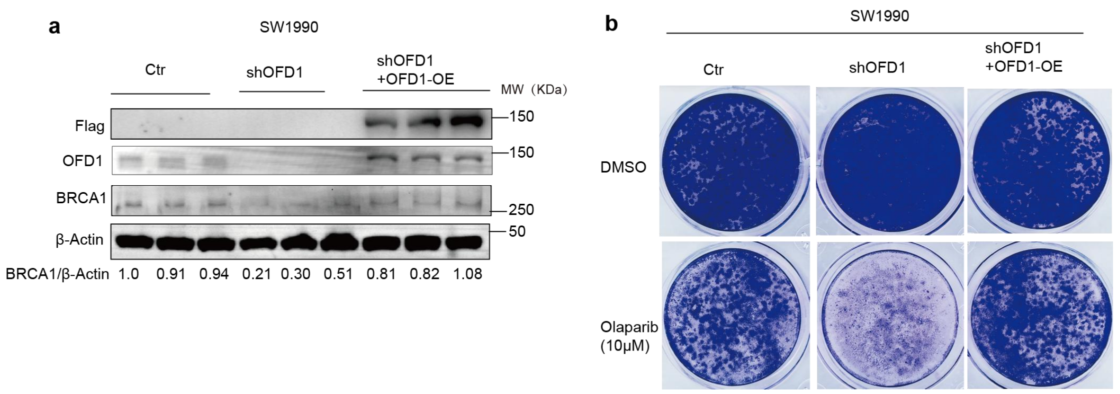

Supplementary Fig. 2 | Combined OFD1 knockdown and olaparib treatment induces a synthetic lethality effect in SW1990 subcutaneous tumor models.

a, Western blot analysis to assess the protein levels of OFD1 and BRCA1 in tumors from each treatment group (Control, shOFD1, and shOFD1 + Flag-OFD1 OE). Flag-Tag was used to detect overexpression of exogenous OFD1. β-Actin was used as a loading control, N = 3 tumor samples. **b**, Colony formation assay of SW1990-derived stable cell lines treated with olaparib. SW1990 cells expressing control shRNA (sh-ctr), OFD1 knockdown (shOFD1), or shOFD1 with OFD1 overexpression (shOFD1 + OFD1-OE) were treated with 10 μM olaparib. Colony formation capacity was assessed to evaluate treatment response.

Supplementary Fig. 3

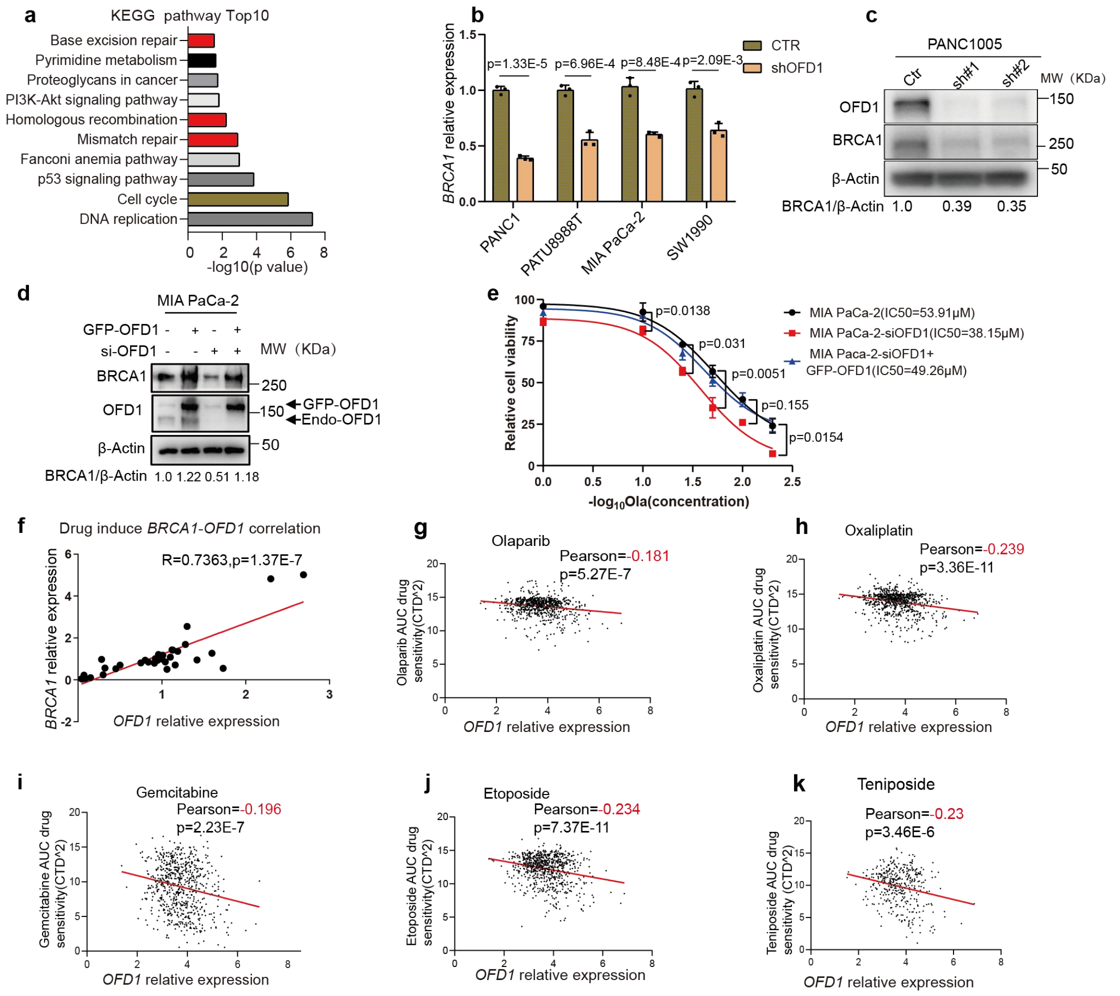

Supplementary Fig. 3 | OFD1 regulates BRCA1 expression and exhibits consistent co-expression patterns across multiple datasets. a, KEGG pathway analysis of the top ten significantly regulated pathways following OFD1 knockdown versus control in PANC1 cells. **b**, Validation of BRCA1 mRNA expression by qPCR following OFD1 knockdown in multiple pancreatic cancer cell lines. Quantification was performed in PANC1, MIA PaCa-2, SW1990, and PATU8988T cells. Data are presented as mean ± SD (n = 3 independent experiments); the statistical analysis was performed using unpaired two-tailed Student's t-test. **c**, Validation of BRCA1 downregulation by western blot in control versus shOFD1 in PANC1005 pancreatic cancer cell lines. Two independent shRNA targeting OFD1 were used. **d**, Rescue of BRCA1 expression by stable reintroduction of siRNA-resistant GFP-OFD1 in MIA PaCa-2 cells. Western blot analysis showed that OFD1 re-expression restored BRCA1 levels reduced by siOFD1 treatment. **e**, Cell viability assays in MIA PaCa-2 cells and derived lines treated with olaparib.

Parental, shOFD1, and shOFD1 + OFD1-OE MIA PaCa-2 cells were treated with increasing concentrations of olaparib for 96 hours. Cell viability was assessed using the CCK8 assay; statistical analysis was conducted using two-way ANOVA followed by Tukey's post hoc test. **f**, Correlation between *OFD1* and *BRCA1* mRNA expression in MIA PaCa-2 treated with different small-molecule inhibitors (listed in **Supplementary Data 4**). Pearson correlation coefficient (two-side) was used for statistical analysis (n = 38 drugs). **g-k**, Correlation between OFD1 mRNA expression and sensitivity to various chemotherapeutic agents in the CTRP database. Correlation between OFD1 expression and drug AUC (area under the dose–response curve) was assessed for olaparib (**g**, n = 756 cell lines), Oxaliplatin (**h**, n = 747 cell lines), Gemcitabine (**i**, n = 690 cell lines), Etoposide (**j**, n = 753 cell lines), and Teniposide (**k**, n = 398 cell lines) using Pearson correlation analysis (two-side).

Supplementary Fig. 4

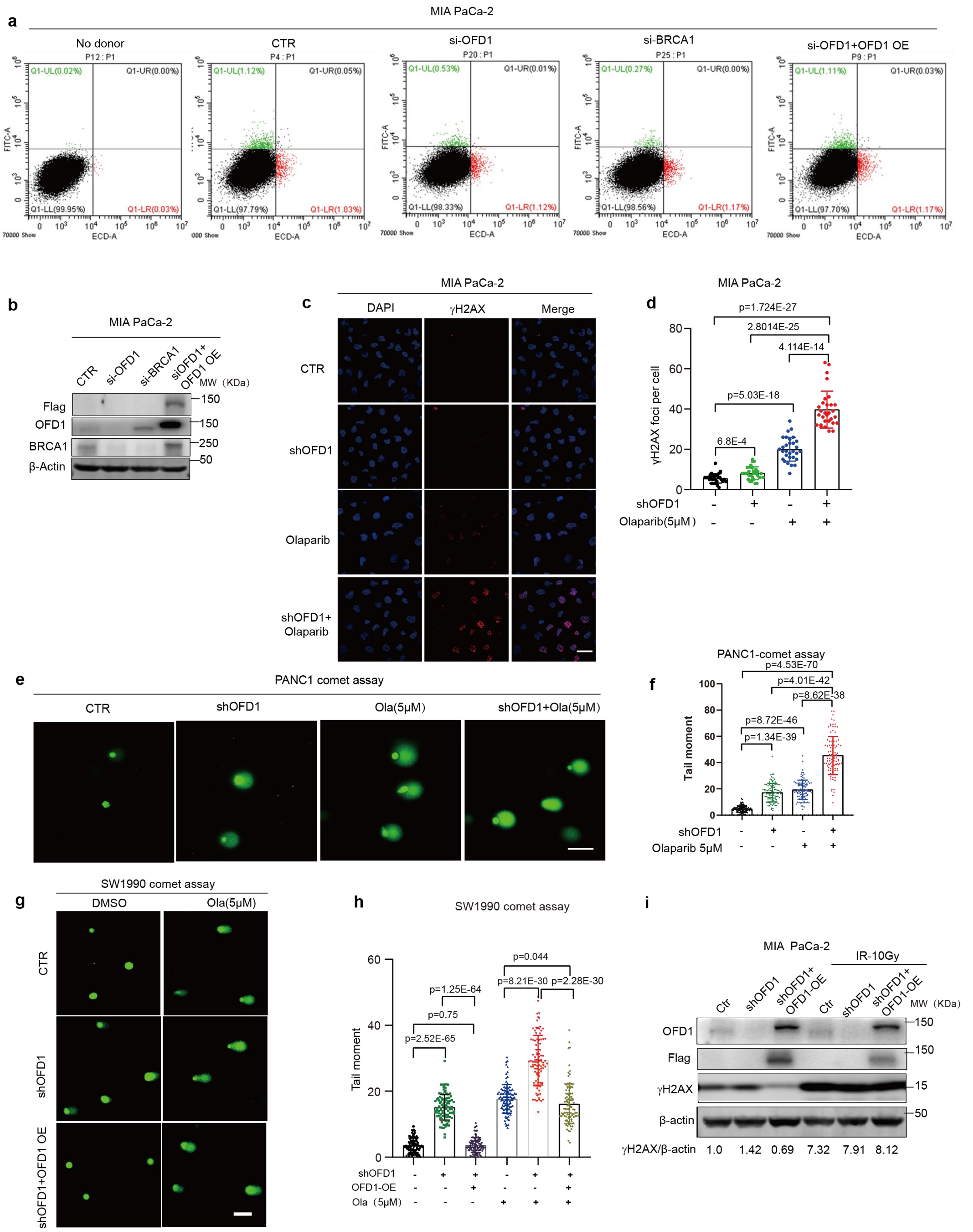

Supplementary Fig. 4 | OFD1 knockdown impairs homologous recombination (HR) repair efficiency upon DNA damage.

a, Quantification of HR and NHEJ repair activity in MIA PaCa-2 DR-GFP cells following OFD1 knockdown. Cells were transfected with si-NC (negative control), si-OFD1, or si-OFD1 + OFD1 overexpression construct (resistant to si-OFD1), followed by I-SceI plasmid transfection. GFP-positive cells represent HR repair events, and mCherry-positive cells indicate NHEJ repair.

si-BRCA1 was used as a positive control for HR deficiency. Analysis was performed by flow cytometry 48 hours post-transfection. **b**, Protein level of OFD1 and BRCA1 were examined by western blot analysis validating knockdown and overexpression efficiency. **c**, Representative immune-fluorescence images showing γH2AX foci in MIA PaCa-2 cells. γH2AX foci indicate the presence of DNA double-strand breaks. Scale bar = 20 μm. **d**, Quantification of γH2AX-positive cells (>10 foci per nucleus) in each treatment group. At least 30 cells per condition were analyzed. Data are presented as mean ± SD. Statistical analysis was performed using unpaired two-tailed Student's t-test. **e**, Neutral comet assay evaluating DNA damage in PANC1 cells with or without OFD1 knockdown treated with 5 μM olaparib or DMSO for 48 hours. Scale bar = 25 μm. **f**, Quantification of tail moment from the comet assay in panel **e** using ImageJ Open Comet software. Data are presented as mean ± SD, with n = 100 cells per group. Experiments were repeated three times. Statistical significance was assessed using unpaired two-tailed Student's t-test. **g**, Neutral comet assay in SW1990 sh-Ctr, shOFD1, and shOFD1 + OFD1 OE cells treated with 0 or 5 μM olaparib for 48 hours. Scale bar = 25 μm. **h**, Comet tail moments quantified using Open Comet software (ImageJ). Data: mean ± SD, 3 biological replicates (100 cells/condition). Comparison by unpaired two-tailed t-test. **i**, Western blot of γH2AX in OFD1-knockdown/re-expressing MIA PaCa-2 cells ± 10 Gy IR. γH2AX levels quantified below as grayscale ratio to β-actin.

Supplementary Fig. 5

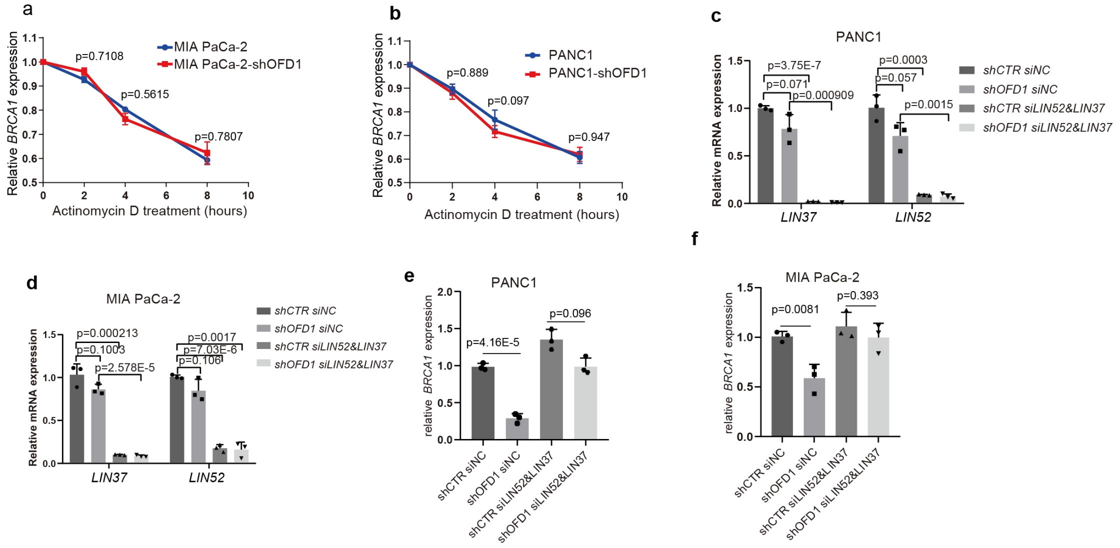

Supplementary Fig. 5| Dissociation of the DREAM complex rescues *BRCA1* downregulation induced by OFD1 knockdown.

a-b, *BRCA1* mRNA stability analysis in MIA PaCa-2 (**a**) and PANC1 (**b**) cells with or without OFD1 knockdown. Cells were treated with the transcriptional inhibitor actinomycin D (5 μg/mL) for 2, 4, 6, and 8 hours. *BRCA1* mRNA levels were quantified by qPCR to assess transcript stability (n = 3 independent experiments). Differences between groups across time points were evaluated by two-way ANOVA followed by Tukey's post hoc test. Error bars represent mean ± SD. **c-d**, Validation of *LIN52* and *LIN37* knockdown efficiency in PANC1 (**c**) and MIA PaCa-2 (**d**) cells. Gene expression was measured by qPCR following siRNA transfection. Data are presented as mean ± SD (n = 3 independent experiments), and statistical significance was determined using unpaired two-tailed Student's t-test. **e-f**, Knockdown of *LIN52* and *LIN37* restores *BRCA1* mRNA expression following OFD1 depletion in PANC1 (**e**) and MIA PaCa-2 (**f**) cells. BRCA1 transcript levels were quantified by qPCR. Data are shown as mean ± SD (n = 3 independent experiments). Statistical comparisons were performed using unpaired two-tailed Student's t-test.

Supplementary Fig. 6

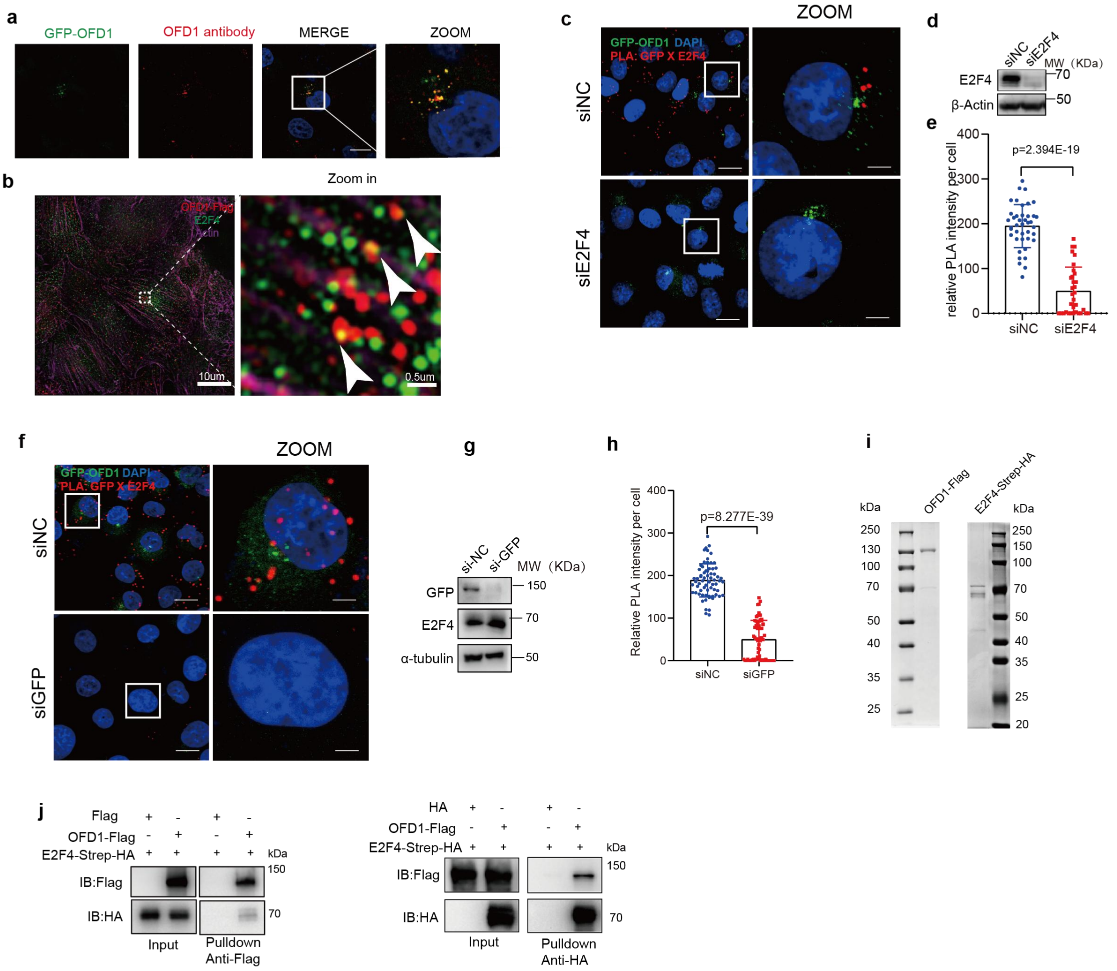

Supplementary Fig. 6| OFD1 interacts with E2F4 to regulate BRCA1 expression.

a, Immunofluorescence analysis confirms that N-terminal GFP-tagged OFD1 co-localizes with endogenous OFD1 in PANC1 cells. **b**, Super-resolution imaging using structured illumination microscopy (SIM) shows co-localization of OFD1-Flag (red) and E2F4 (green). Actin filaments were labeled with Brilliant Violet dyes as cytoskeletal markers. Scale bar = 10 μm. **c-h**, Co-localization of OFD1 and E2F4 in PANC1 cells visualized by proximity ligation assay (PLA) assay. **c**, Representative images and zoomed-in pictures showing the co-localization of OFD1 and E2F4 in PANC1 cells with or without E2F4 knockdown. Scale bar = 10 μm, Zoom in Scale bar = 2.5 μm. **d**, Validation of E2F4 knockdown in PANC1 by Western blot. **e**, The PLA signal intensity in each cell was quantified by ImageJ and presented. N =33 cells in siNC group and n = 41 cells in siE2F4 group, unpaired two tailed student's t-test. **f**, Representative images and zoomed-in pictures showing the co-localization of OFD1 and E2F4 in PANC1 cells with or without GFP

knockdown, visualized by the PLA assay. Scale bar = 10 μm, Zoom in Scale bar = 2.5 μm. **g**, Validation of GFP knockdown in PANC1 by western blot. **h**, Quantification of PLA signal intensity in each cell using ImageJ. Scale bar = 10 μm. Zoom in Scale bar = 2.5 μm. Error bars, n = 65 cells in siNC group and n = 68 cells in siGFP group, mean ± SD, unpaired student's t-test (two-tailed). **i**, Protein purification of recombinant C-terminal Flag-tagged OFD1 and C-terminal tandem Strep-HA-tagged E2F4. **j**, *In vitro* pulldown assay confirms a direct protein–protein interaction between recombinant OFD1 and E2F4.

Supplementary Fig. 7

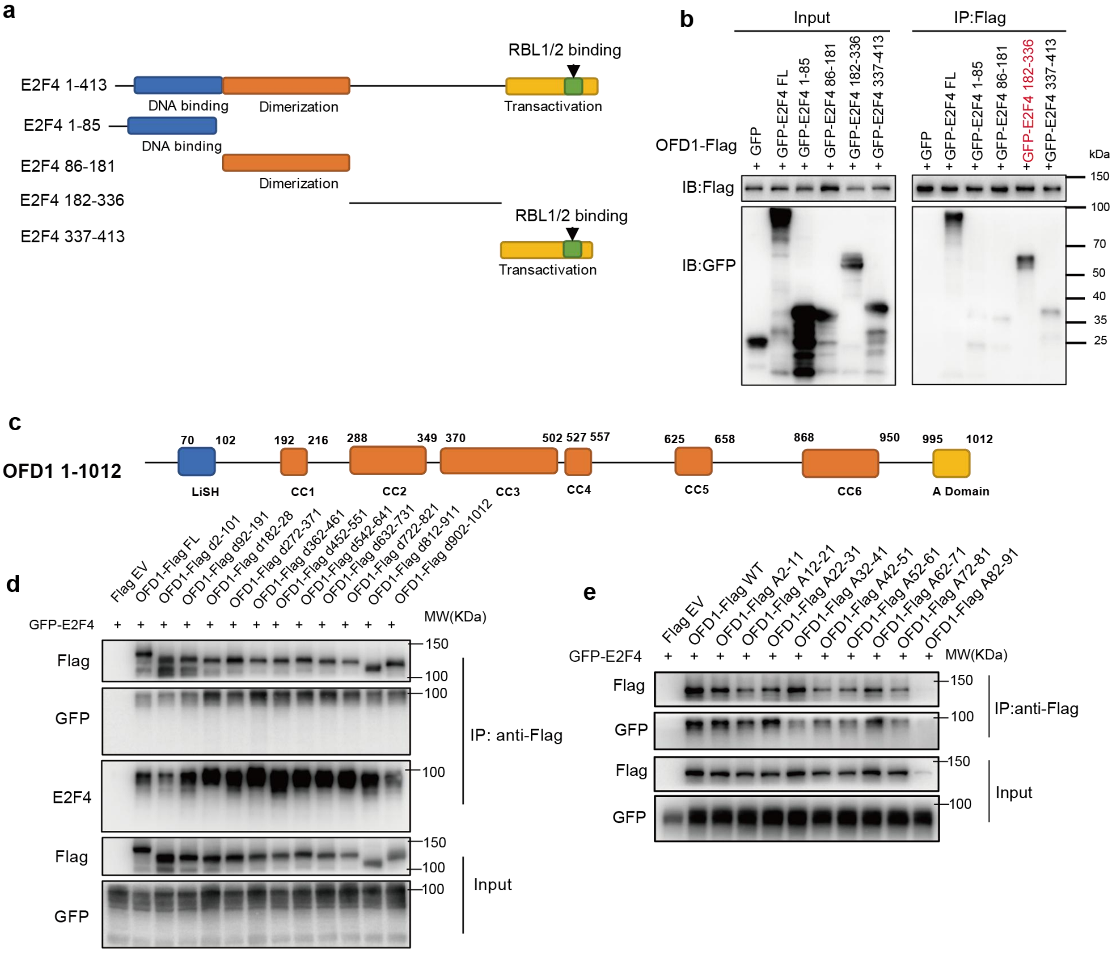

Supplementary Fig. 7 | Domain mapping of the interaction between OFD1 and E2F4.

a, Schematic diagram showing full-length and truncated E2F4 proteins based on known functional domains, with the regulatory domain (amino acids 182-336) highlighted. **b**, Immunoprecipitation assay demonstrating that OFD1 primarily interacts with the regulatory domain of E2F4 (182–336 aas). **c-e**, Domain mapping of the OFD1-E2F4 interaction. **c**, Domain structures of OFD1 protein. **d**, Co-immunoprecipitation showing that E2F4 binds to the 2-101 amino acids of OFD1. **e**, Co-IP results showing that 32-41 amino acids of OFD1 are essential for E2F4 binding.

Supplementary Fig. 8

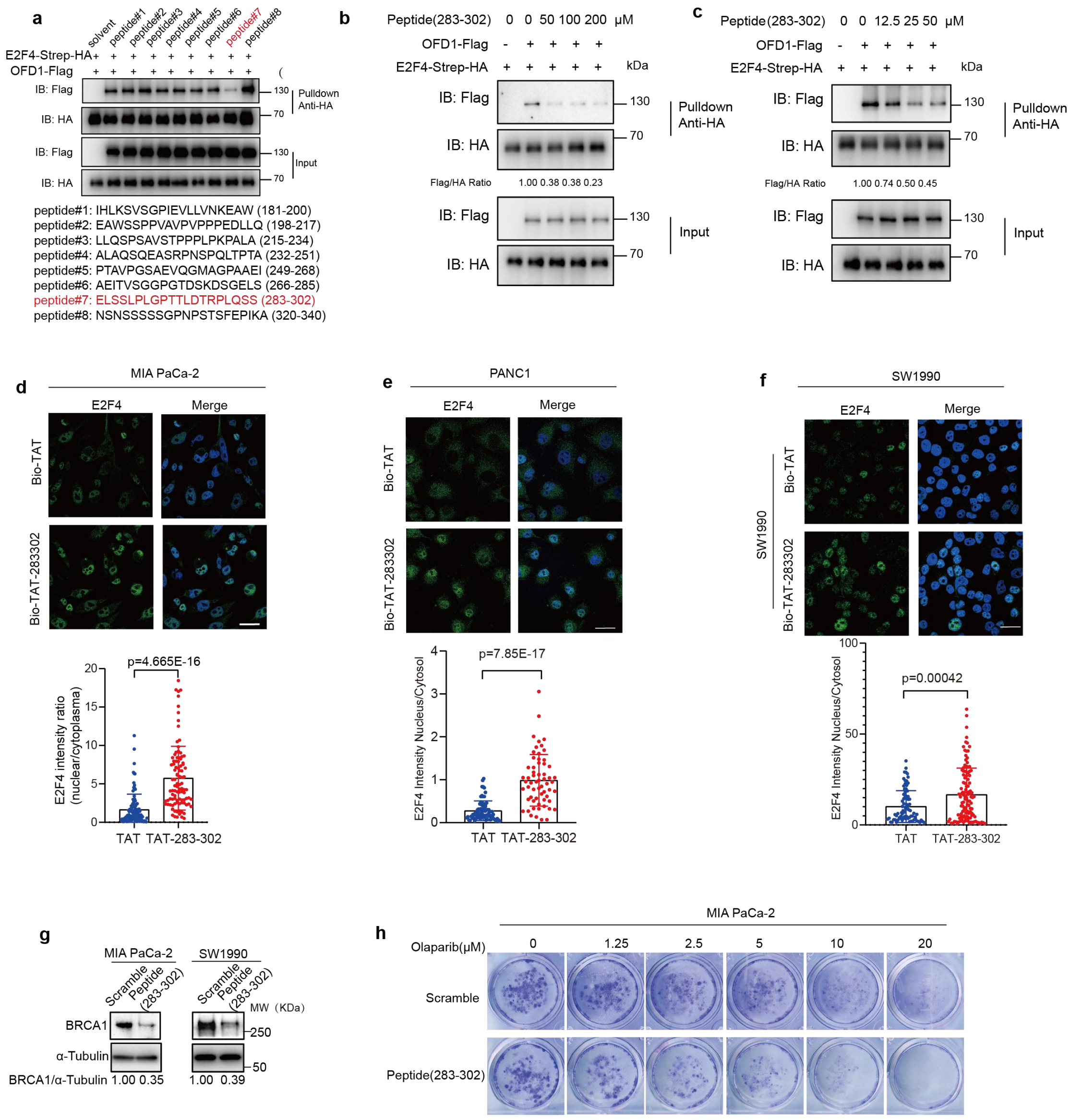

Supplementary Fig. 8| The effect of a peptide disrupting OFD1-E2F4 interaction and mimicking OFD1 knockdown.

a, *In vitro* peptide competition screening identified the precise OFD1-binding motif within E2F4. The interaction was mapped to amino acids 283–302 of E2F4. The sequences of candidate peptides derived from the E2F4 regulatory domain are shown; the competitive peptide is highlighted in red. **b-c**, *In vitro* pulldown assays evaluating the binding between OFD1 and E2F4 in the presence of the competitive peptide (283–302) at titrated concentrations ranging from 0 to 200 µM (**b**) and 0 to 50 µM (**c**). **d-f**, Representative immunofluorescence (IF) images showing the nuclear translocation of E2F4 in three pancreatic cancer cell lines (MIA PaCa-2, PANC1, SW1990) treated with 25 µM competitive peptide (283–302) or control peptide for 48 hours. Quantification

of the nuclear-to-cytoplasmic E2F4 intensity ratio in MIA PaCa-2 (**d**), PANC1 (**e**), and SW1990 (**f**) cells. Scale bar = 10 μm. Error bars represent mean ± SD, MIA PaCa-2 Bio-TAT: n = 100 cells, MIA PaCa-2 Bio-TAT-283302: n = 97 cells, PANC1 Bio-TAT: n = 77 cells, PANC1 Bio-TAT-283302: n = 61 cells, SW1990 Bio-TAT: n = 81 cells, SW1990 Bio-TAT-283302: n = 116 cells, unpaired two-tailed Student's t-test. **g**, Western blot analysis of BRCA1 expression in MIA PaCa-2 cells treated with 25 μM peptide (283–302) or control peptide for 48 hours. **h**, Colony formation assay assessing the effect of the 25 μM peptide (283–302) in combination with varying concentrations of olaparib (0, 1.25, 2.5, 5, 10, 20 μM) in MIA PaCa-2 cells.

Supplementary Fig. 9

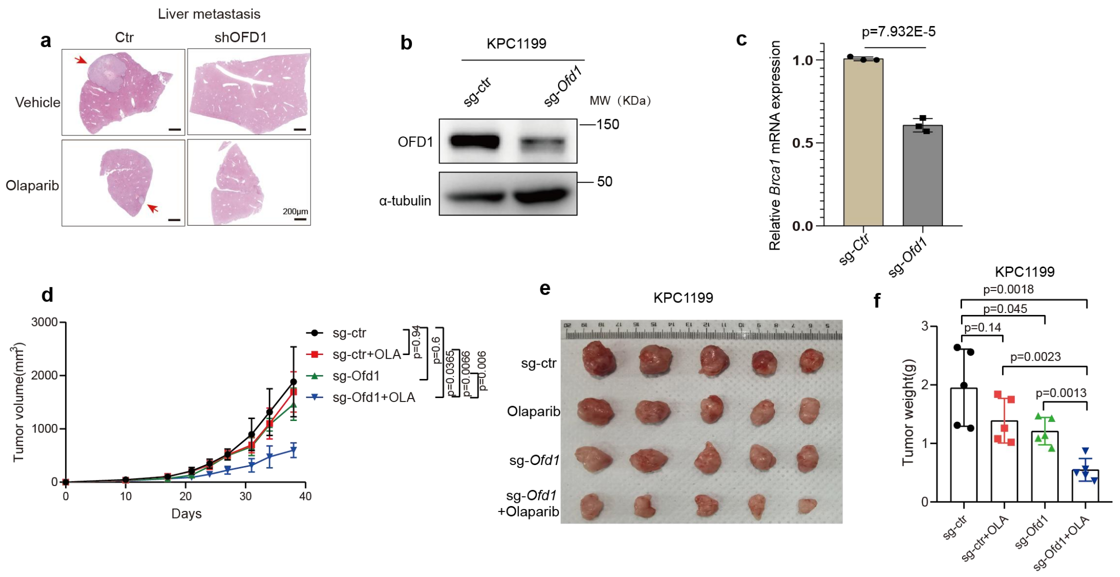

Supplementary Fig. 9| The combination of OFD1 knockdown and PARPi effectively suppresses tumor growth in a KPC1199 xenograft model.

a, Representative H&E staining images showing liver metastasis burden at the experimental endpoint in MIA PaCa-2 orthotopic pancreatic cancer models. Scale bar = 200 µm. **b**, Western blot analysis of OFD1 protein levels in wild-type (WT) and Ofd1-knockout (Ofd1-KO) KPC1199 pancreatic cancer cell lines. **c**, *Brca1* mRNA expression was assessed by qPCR in *WT* and *Ofd1-KO* KPC1199 pancreatic cell lines. Error bars, mean ± SD, n = 3 independent experiments, unpaired two-tailed Student's t-test. **d-f**, Evaluation of tumor growth in KPC1199-sgCtrl and KPC1199-sgOfd1 xenograft models treated with vehicle or olaparib. Tumor volumes (mm^3) were monitored over time and analyzed by two-way ANOVA followed by Tukey's post hoc test (**d**, n = 5 tumors per group). Representative tumor images from each group were taken at day 38 of treatment (**e**). Final tumor weights were measured at the end of treatment as an indicator of tumor burden (**f**). Error bars represent mean ± SD, n = 5 tumors, unpaired two-tailed Student's t-test.

Supplementary Fig. 10

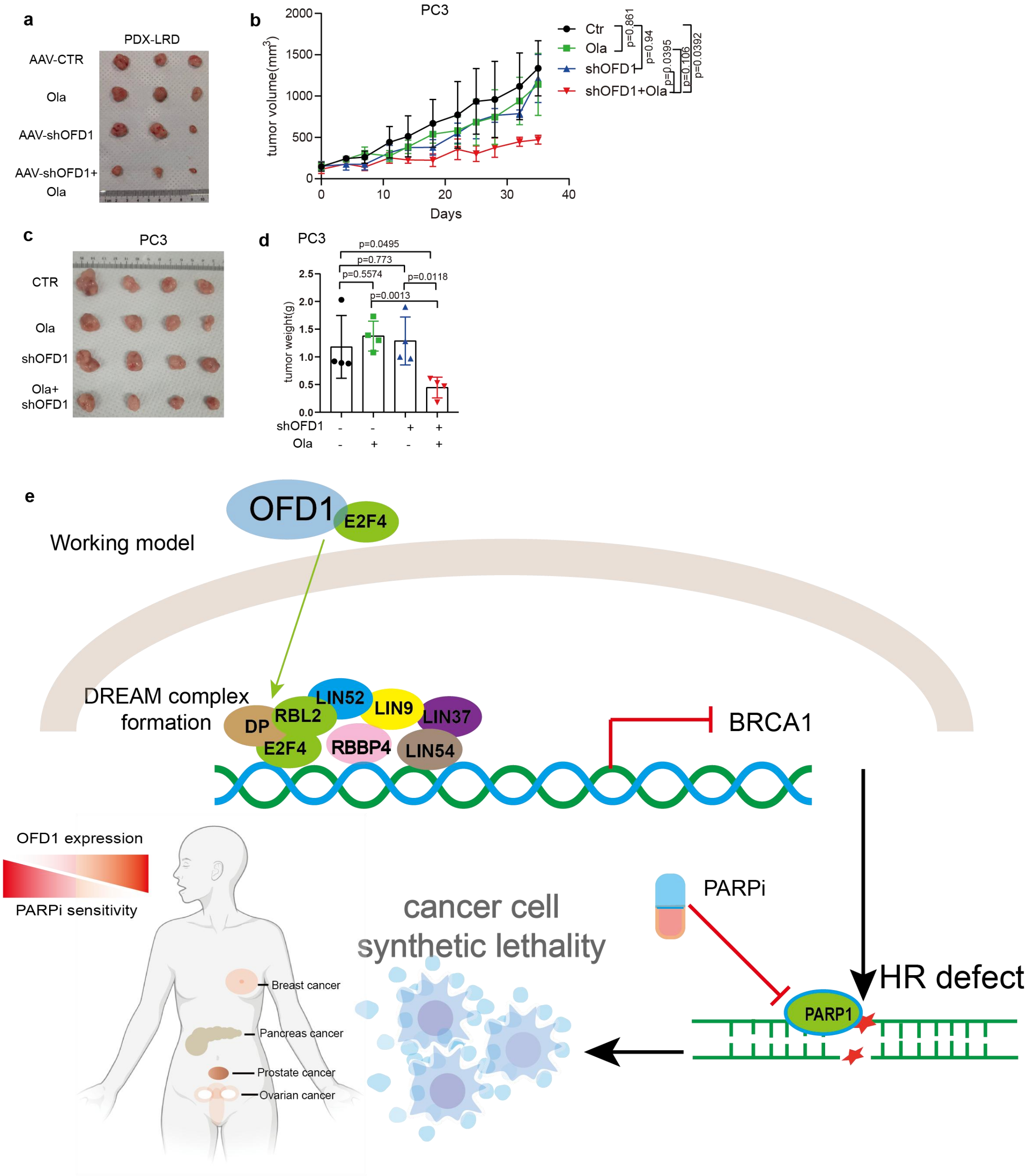

Supplementary Fig. 10| OFD1 Knockdown–PARPi Synergy in PDAC and Prostate Cancer and Proposed Mechanism. a, Representative images of harvested tumors from each treatment group (n = 3 mice) in the PDX-LRD model. Xenograft models were treated with olaparib, AAV-shOFD1, or a combination treatment. **b-d**, Tumor volume (mm^3) measurements in PC3 and PC3-shOFD1 xenograft models treated with vehicle or olaparib. The significant differences in tumor volume between the treatment groups were analyzed by two-way ANOVA followed by Tukey's post hoc test (**b**, n = 4 mice per group). Representative images of tumors collected and photographed after 35 days of treatment (**c**, n = 4 tumors per group). Tumor weight was measured

as a surrogate for tumor burden at the end of treatment. (**d**). Error bars represent mean ± SD, n = 4 tumors, unpaired two-tailed Student's t-test. (**e**) Working model: OFD1 sequesters E2F4 in the cytosol, preventing DREAM-mediated BRCA1 repression. Inhibiting OFD1 restores DREAM complex formation at the BRCA1 promoter, enhancing sensitivity to Olaparib in BRCA-associated tumors (Created in BioRender. Li, P. (2025) https://BioRender.com/5p8gqcg).

Supplementary Table 1. Materials used in this study

Antibodies

**Antibodies**	Source	Identifier
Rabbit polyclonal anti-OFD1 antibodies	Cao et al.,2023	
Mouse monoclonal alpha-tubulin antibodies	Abcam	Cat# ab7291; RRID: AB_2241126
Mouse monoclonal beta-Actin antibodies	Abcam	Cat# ab 8226; RRID: AB_306371
Rabbit polyclonal anti-BRCA1 antibodies	Cell signaling technology	Cat#9010S; RRID: AB_2228244
Mouse monoclonal anti-BRCA1 antibodies	Santa Cruz	Cat#sc-6954; RRID: AB_626761
Rabbit polyclonal anti-E2F4 antibodies	Cell signaling technology	Cat# #40291 RRID: AB_2799174
Rabbit polyclonal anti-E2F1 Antibodies	Cell signaling technology	Cat #3742 RRID: AB_2096936
Rabbit polyclonal anti-RBL2 Antibodies	Cell signaling technology	Cat # 13610 RRID: AB_2798274
Rabbit polyclonal anti-RBBP4 Antibodies	Abclonal	Cat #A3645; RRID: AB_2863104
Rabbit polyclonal anti-LIN54 Antibodies	Bethyl Laboratories	Cat #A303-799A; RRID: AB_11218173
Rabbit polyclonal anti-Ofd1 antibodies	Home made	
Rabbit polyclonal anti-KI67 antibody	Proteintech	Cat #27309-1-AP; RRID: AB_2918145
Mouse monoclonal anti-Flag antibody	Merck	Cat #F1804; RRID: AB_262044
Mouse monoclonal GFP tag antibody	Proteintech	Cat #66002-1-Ig; RRID: AB_11042881
Rabbit polyclonal anti-γH2A.X antibodies	Cell signaling technology	Cat #60566;

Biological samples

Biological samples	Source
Tissue specimens of patients with PDAC	Shanghai Outdo Biotech Co. Ltd
PDAC PDX tumor	Ruijin Hospital, Shanghai, China

Chemicals, reagents

**Chemicals, reagents**	**Source**	**Identifier**

Olaparib	TOPSCIENCE	Cat#T3015
Harmine	TOPSCIENCE	Cat#T1711
PEG300	MedChemExpress	Cat# HY-Y0873
Penicillin/Streptomycin	Invitrogen	Cat# 15140122
HEPES	Sigma-Aldrich	Cat#H3375
Triton X-100	Sigma-Aldrich	Cat# T8787
CCK8	DOJINDO	Cat#CK04
Crystal violet	Sangon Biotech	Cat#A600331
Blasticidin	Invivogen	Cat#ant-bl-05
Puromycin	Invivogen	Cat#ant-pr-1
SYBR Green PCR Master Mix	Vazyme	Cat#Q111-02
b-mercaptoethanol	Millipore	Cat#ES-007-E
Matrigel	Corning	Cat#356234
Protein G Magnetic Beads	Cell signaling technology	Cat##9006

Critical commercial assays

Reagent or resource	Source (Company)	Identifier
Comet Assay Kit	Abcam	Cat#ab238544
Chromatin immunoprecipitation (ChIP) kit	Merck	Cat#17-408
VivoGlo™ Luciferin, In Vivo Grade	Promega	Cat#P1043
FastPure Cell/Tissue Total RNA Isolation Kit V2	Vazyme	Cat#RC112-01

Experimental models: Cell lines

Human: MIA PaCa-2 cells	ATCC	Cat# CRL-1420
Human: BxPC-3 cells	ATCC	Cat# CRL-1687
Human: HPAC cells	ATCC	Cat# CRL-2119
Human: SW1990 cells	ATCC	Cat#CRL-2172
Human: PANC1 cells	ATCC	Cat#CRL-1469
Human: PATU8988T cells	DSMZ	Cat# ACC 162
Human: PANC1005 cells	ATCC	Cat#CRL-2547
Human: MDA-MB231 cells	ATCC	Cat#HTB-26
Human PC3 cells	ATCC	Cat#CRL-1435
Human A2780 cells	ATCC	
Human HPAFII cells	ATCC	Cat#CRL-1997
Mouse: KPC derived PDAC cell line KPC1199	Hingorani et al.2005	

Experimental models: mouse strains

Resource	Source	Identifier
Mouse: *BALB/c-nu*	Shanghai Lingchang biotech	
Mouse: *LSL-Kras* $^{G12D/+}$	Jackson Laboratories	Stock#008179IMSR_JAX:008179
Mouse: *Trp53*$^{R172H/+}$	Jackson Laboratories	Stock#008652IMSR_JAX:008652
Mouse: *Pdx1-Cre*	Jackson Laboratories	Stock#014647IMSR_JAX:014647
Mouse: *Ofd1(fl/fl)*	Shanghai Model Organisms Center, Inc.	

Supplementary Table 2. Small molecule synthetic lethality screen targeting OFD1 in MIA PaCa-2 cells

Category	Parameter	Description
Assay	Type of assay	In vitro, cell-based assay
	Target	Synthetic lethal interaction with OFD1 knockdown
	Primary measurement	Cell viability (measured by CCK-8 assay, absorbance at 450 nm)
	Key reagents	Selleck Chemicals FDA-approved Drug Library (Cat# L1300), Doxycycline (100 ng/mL), Cell Counting Kit-8 (CCK-8, Dojindo), MIA PaCa-2-tet-on-shOFD1 cell line<br><br>DMEM + 10% FBS + 1% penicillin/streptomycin
	Assay protocol	A high-throughput, in vitro cell-based screening assay was conducted to identify small-molecule compounds that exhibit synthetic lethality with OFD1 knockdown in human pancreatic cancer cells. A selected subset of anti-cancer compounds from the FDA-approved drug library (Selleck Chemicals, Cat# L1300) was screened in this assay. Human pancreatic cancer cells MIA PaCa-2-tet-on-shOFD1, which stably express doxycycline-inducible shRNA targeting OFD1 under a Tet-on system, were maintained in Dulbecco's Modified Eagle Medium (DMEM) supplemented with 10% fetal bovine serum (FBS) and 1% penicillin-streptomycin. Cells were seeded at a density of 2000 cells per well in 96-well tissue culture plates with 100 µL of complete medium per well and allowed to attach overnight. Cells were then divided into two treatment groups: Control group: cells were treated with DMSO only and no doxycycline, maintaining endogenous OFD1 expression. Knockdown group: cells were treated with 100 ng/mL doxycycline to induce OFD1 knockdown. Both groups were treated in parallel with the selected anti-cancer small-molecule compounds at six concentrations: 0 µM, 1 µM, 5 µM, 10 µM, 25 µM, and 50 µM. DMSO concentration was kept constant across all wells to ensure consistency. Each condition was performed in at least three technical replicate wells. Plates were incubated under standard tissue culture conditions (37 °C, 5% CO_2) for 72 hours. After treatment, cell viability was assessed using the Cell Counting Kit-8 (CCK-8; Dojindo) according to the manufacturer's protocol. CCK-8 reagent (10 µL) was added to each well and incubated for 2 hours before measuring absorbance at 450 nm using a microplate reader. For compounds exhibiting low IC50 values, additional concentrations around the estimated IC50 were included at lower dose ranges to enhance resolution. Conversely, for compounds with higher IC50 values, supplementary higher concentrations were incorporated to ensure accurate curve fitting and reliable IC50 estimation. This approach ensured better coverage of the IC50 range for more accurate estimation.<br>Dose–response curves were generated using GraphPad Prism 8 (nonlinear regression, four-parameter logistic model, bottom constrained to 0, top constrained to 1). LogIC50 values were calculated for each condition. To identify compounds with significant synthetic lethal effects, extra sum-of-squares F tests were performed comparing the knockdown and control dose–response fits.
	Additional comments	

Library	Library size	217 compounds
	Library composition	FDA-approved small-molecule compounds with known or potential anti-cancer activity, selected from the Selleck Chemicals L1300 library. The subset includes diverse classes of oncology drugs targeting kinases, epigenetic regulators, apoptosis pathways, and DNA damage response.
	Source	Selleck Chemicals, FDA-approved Drug Library (Cat# L1300)
	Additional comments	The screening was performed using a doxycycline-inducible Tet-on shRNA system to achieve OFD1 knockdown in MIA PaCa-2 cells.<br><br>Each compound was tested at six concentrations with at least three technical replicates per condition.<br><br>Cell viability was measured after 72 hours of treatment using the CCK-8 assay.<br><br>The goal was to identify compounds exhibiting synthetic lethality with OFD1 depletion.<br><br>DMSO-treated cells without doxycycline served as controls to distinguish the effect of OFD1 knockdown
Screen	Format	n vitro cell-based screening performed in 96-well plates with doxycycline-inducible shOFD1 MIA PaCa-2 cells
	Concentration(s) tested	Six concentrations tested per compound: 0, 1, 5, 10, 25, and 50 micromolar (μM)
	Plate controls	DMSO-treated MIA PaCa-2-tet-on-shOFD1 cells without doxycycline induction as control wells.
	Reagent/ compound dispensing system	Manual pipetting using multichannel pipettes
	Detection instrument and software	PerkinElmer multimode plate reader; data analyzed using GraphPad Prism 8
	Assay validation/QC	Each condition was performed in at least three technical replicates to ensure reproducibility.<br>Dose-response curves were generated and analyzed using nonlinear regression with variable slope (four-parameter logistic model).<br>LogIC50 values were statistically compared using the extra sum-of-squares F Test to assess significance.<br>Consistent cell seeding density and viability measurement protocols were maintained across all plates.<br>DMSO-treated, non-induced cells served as controls to monitor baseline viability and assay consistency.
	Correction factors	Background absorbance from blank wells (media without cells) was subtracted from all readings to correct for non-specific signal.<br>For compounds exhibiting low IC50 values, additional concentrations around the estimated IC50 were included at lower dose ranges to enhance resolution. Conversely, for compounds with higher IC50 values, supplementary higher concentrations were incorporated to ensure accurate curve fitting and reliable IC50 estimation. This approach ensured better coverage of the IC50 range for more accurate estimation.
	Normalization	Cell viability values were normalized to DMSO-treated control wells without doxycycline to calculate relative viability.
	Additional comments	
Post-HTS analysis	Hit criteria	Top 10 compounds showing greatest synthetic lethality, defined by the highest percentage difference in IC50 between MIA PaCa-2 and MIA PaCa-2-tet-on-shOFD1 cells, calculated as:

	IC50 difference (%) = ((IC50_MIA - IC50_MIA-shOFD1) / IC50_MIA) * 100%
Hit rate	Approximately 4.6% (10 hits out of 217 compounds tested)
Additional assay(s)	Follow-up validation of hit compounds was performed by dose-response assays in MIA PaCa-2-tet-on-shOFD1 cells with doxycycline induction to confirm synthetic lethality. Cell viability was assessed using CCK-8 assay at 72 hours post-treatment. To improve IC50 estimation, lower concentrations were added for compounds with low IC50 values to enhance resolution, while higher concentrations were included for those with high IC50 values to ensure accurate curve fitting.
Confirmation of hit purity and structure	Compounds were obtained from the Selleck Chemicals FDA-approved library (Cat# L1300), which provides quality-controlled, commercially sourced compounds with verified purity and structure. No additional purity confirmation was performed in-house.
Additional comments	NO

Supplementary Table 3. PDX patient information table

Gender	Age (years)	Pathological classification	Tumor Location	Histological Grade	Non-synonymous mutations in the exonic regions of BRCA1/2 genes
Female	50-55	pancreatic ductal carcinoma	Distal Pancreas	Ductal Adenocarcinoma, Moderately-Low Differentiation	None
Male	70-75	pancreatic ductal carcinoma	Head of the Pancreas	Ductal Adenocarcinoma, Moderately Differentiated	None

Supplementary Table 4. Oligonucleotide sequences used in this paper.

Sequences	SOURCE	IDENTIFIER
ShOFD1 #1 (sense)<br>5′-GAACGAAGAGAACTAGAAA-3′	This paper	N/A
ShOFD1 #2 (sense)<br>5′-CGAAAAGGCTATAGTGGTT-3′	This paper	N/A
siRNA ARP2 (sense)<br>5′-ACGGUUGGAACGAGAACUUAA-3′	This paper	N/A
SgRNA Control (sense)<br>5′-GAAATGCTATGCTTCGGTTC-3′	This paper	N/A
SgRNA Ofd1 (sense)<br>5′-GACCG GTTAGAAGCCCCTATTAGGA-3′	This paper	N/A
SgRNA E2F4 (sense)<br>5′-CAGAAGGGACACGAACTTGG-3′	This paper	N/A
siRNA OFD1 #1 (sense)<br>5′-GAACGAAGAGAACTAGAAA-3′	This paper	N/A
siRNA OFD1 #2 (sense)<br>5′-CGAAAAGGCTATAGTGGTT-3′	This paper	N/A
siRNA LIN37 #1 (sense)<br>5′-CACUCAUCUAUCGCAACAUTT-3′	This paper	N/A
siRNA LIN37 #2 (sense)<br>5′-GUCGUGAUGUGUACAAGCUTT-3′	This paper	N/A
siRNA LIN52 #1 (sense)<br>5′-GGCUGAGAUAGAACGUGAUTT-3′	This paper	N/A
siRNA LIN52 #2 (sense)<br>5′-GCCAGAACAAUUACCAGGUTT-3′	This paper	N/A
siRNA BRCA1 #1 (sense)<br>5′-GCAGCAGUAUAAGCAAUAUTT-3′	This paper	N/A
siRNA BRCA1 #2 (sense)<br>5′-GCUCCAGUAUUAAUGAAAUTT-3′	This paper	N/A
siRNA E2F4 #1 (sense)<br>5′-GAGAUACCCUCUUGGCCAUTT-3′	This paper	N/A

siRNA E2F4 #2 (sense)<br>5′-GCAGAUGACUACAACUUUATT-3′	This paper	N/A
siRNA GFP #1 (sense)<br>5′-AAGCUGACCCUGAAGUUC-3′	This paper	N/A
siRNA GFP #2 (sense)<br>5′-GGCACAAGCUGGAGUACAA-3′	This paper	N/A

RT-qPCR primers

Sequences	SOURCE	IDENTIFIER
OFD1 #1<br>F: 5′-ACCAGACGTTTAAGGATCGGG-3′<br>R: 5′-GTTCTCCACTCAATACAGGG-3′	This paper	N/A
OFD1 #2<br>F: 5′-AGCCCAGTCTTTGGCAATAAC-3′<br>R: 5′-GGAGACGCAGGTTTTCATTTCT-3′	This paper	N/A
BRCA1<br>F: 5′-GCCAAGGCAAGATCTAGAGG-3′<br>R: 5′-GTTGCCAACACGAGCTGA-3′	This paper	N/A
GAPDH<br>F: 5′-ACAACTTTGGTATCGTGGAAGG-3′<br>R: 5′-GCCATCACGCCACAGTTTC-3′	This paper	N/A
LIN37<br>F: 5′-ACTGGCAAAAGGCCATCTG-3′<br>R: 5′-CTCCGGTCGAACAGCTTGA-3′	This paper	N/A
LIN52<br>F: 5′-CTAGTTCTCCACCCAAATGGATG-3′<br>R: 5′-GCTGATAGGCTAGGTTCTGTAGG-3′	This paper	N/A
Ofd1 #1<br>F: 5′-CCCCACAAGTCTGATGTGTTG-3′<br>R: 5′-TCGGAGTTGGGTCTGAAGTGT-3′	This paper	N/A
Ofd1 #2<br>F: 5′-AGGATCGGGGTGTACTGGAC-3′<br>R: 5′-CTTCTCCACTCAATACAGGATGC-3′	This paper	N/A
Brca1 #1<br>F: 5′-CGAATCTGAGTCCCCTAAAGAGC-3′<br>R: 5′-AAGCAACTTGACCTTGGGGTA-3′	This paper	N/A
Brca1 #2<br>F: 5′-CTGCCGTCCAAATTCAAGAAGT-3′<br>R: 5′-CTTGTGCTTCCCTGTAGGCT-3′	This paper	N/A

ChIP-qPCR primers.

Sequences	SOURCE	IDENTIFIER
BRCA1<br>F: 5′-TTCTTCCTCTTCCGTCTCTTTCC-3′<br>R: 5′-GAGCTCACGCCGCGCAGT-3′	This paper	N/A
ACTB<br>F: 5′-CGGCCAACGCCAAAACT-3′<br>R: 5′-CCCTCTCCCCTCCTTTTGC -3′	This paper	N/A

Gating strategy for HR reporter assay by FACS.

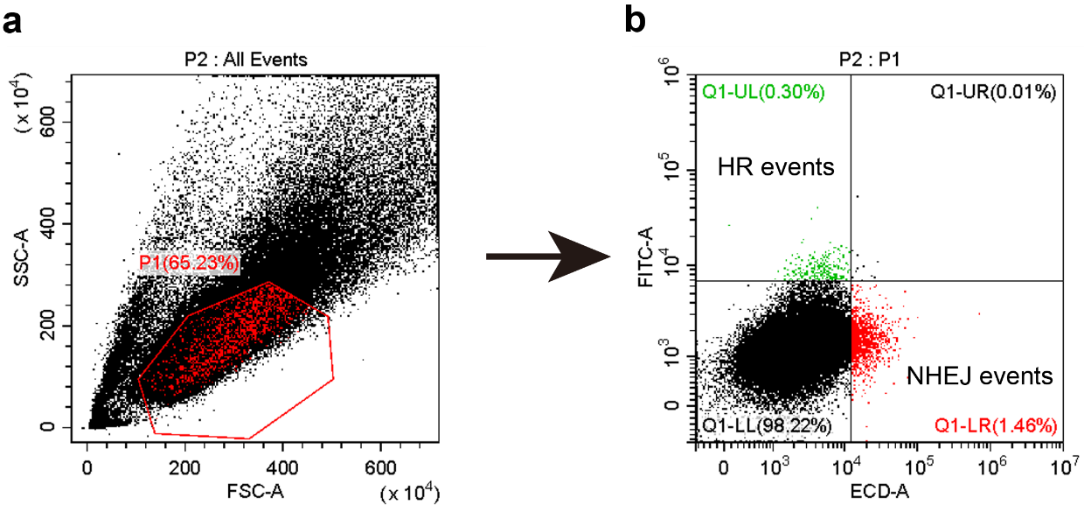

(a) Forward scatter area (FSC-A) and forward scatter height (FSC-H) were used to identify single cells.

(b) FITC (emission wavelength: 519 nm) was utilized to label GFP-expressing cells (HR events), while ECD (emission wavelength: 615 nm) was employed to label mCherry-expressing cells (NHEJ events).

Source Data of Supplementary Figures

Supplementary Fig 2. a

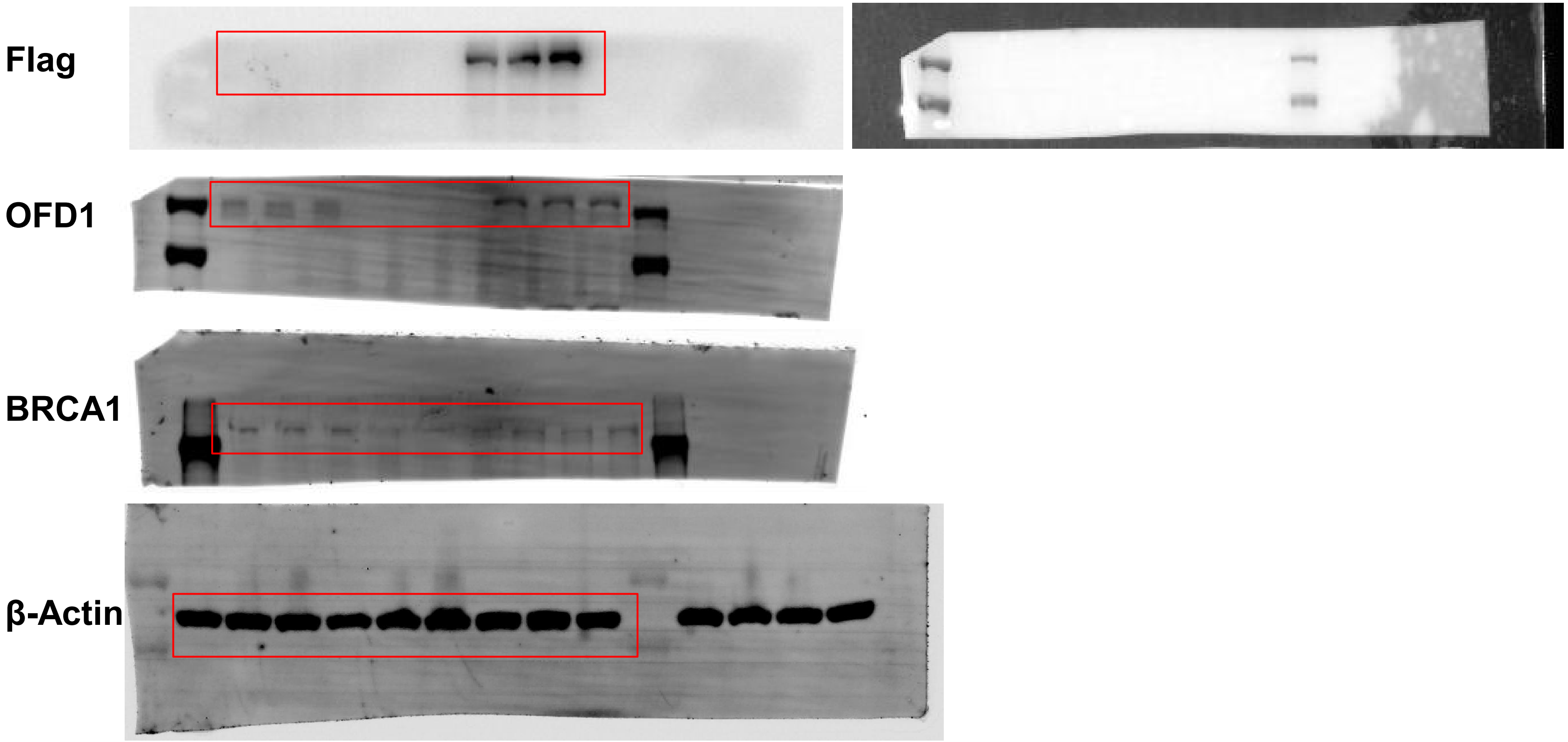

Supplementary Fig 3. c

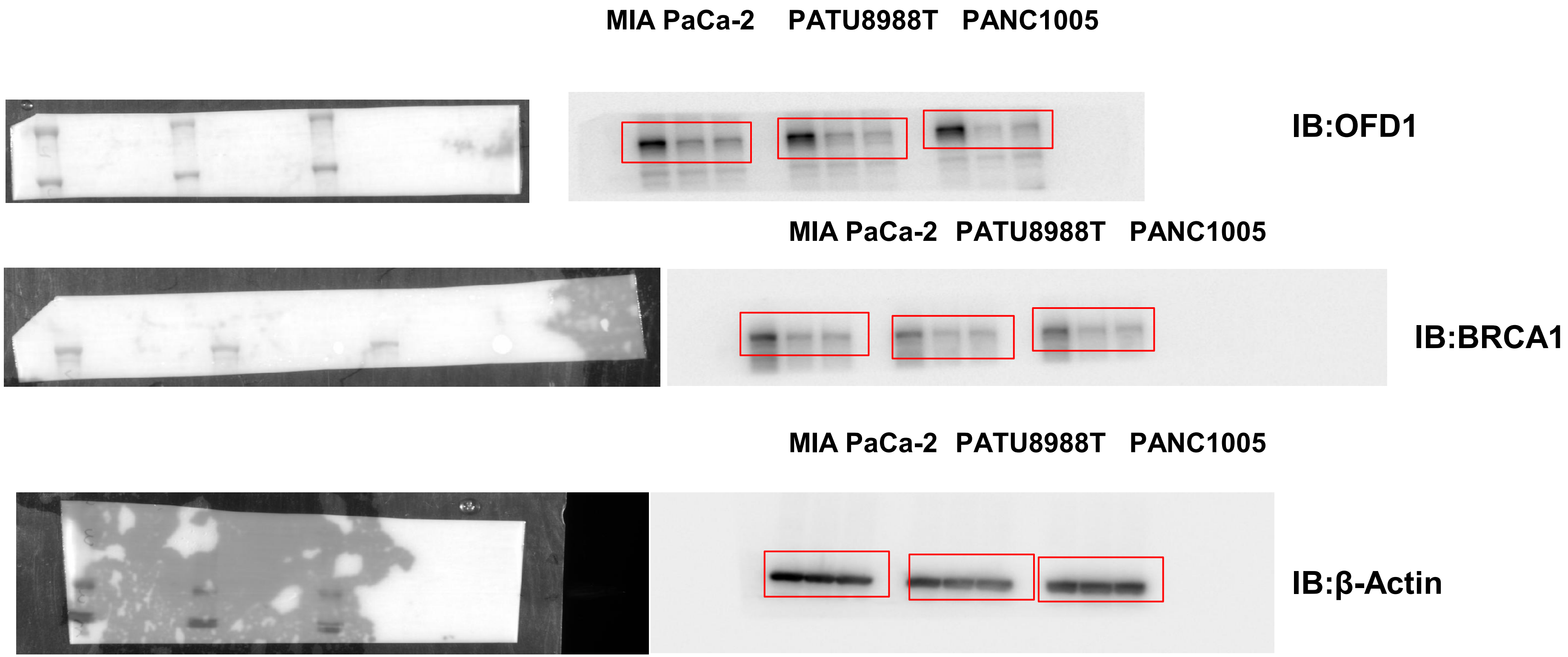

Supplementary Fig 3. d

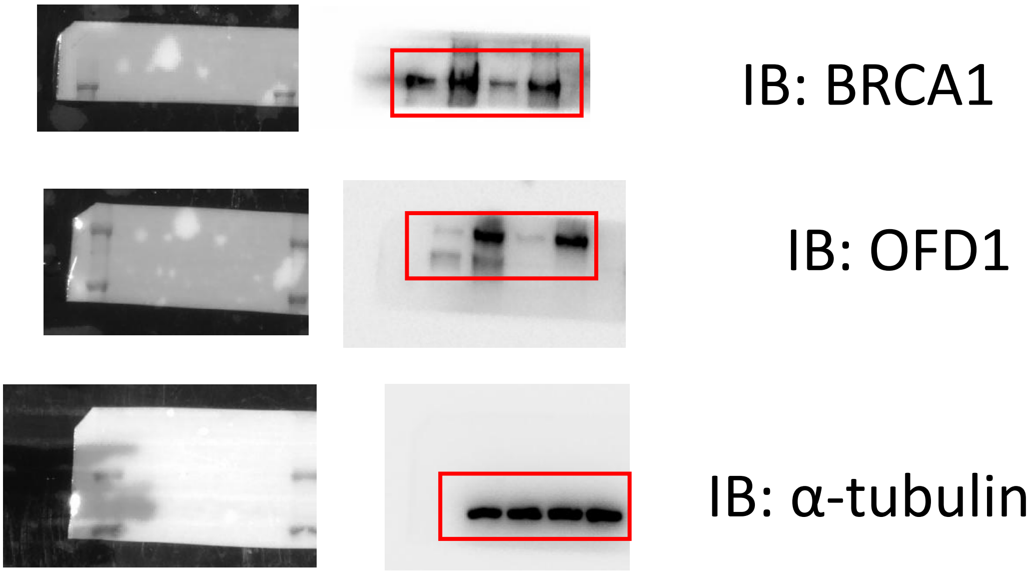

Supplementary Fig 4. b

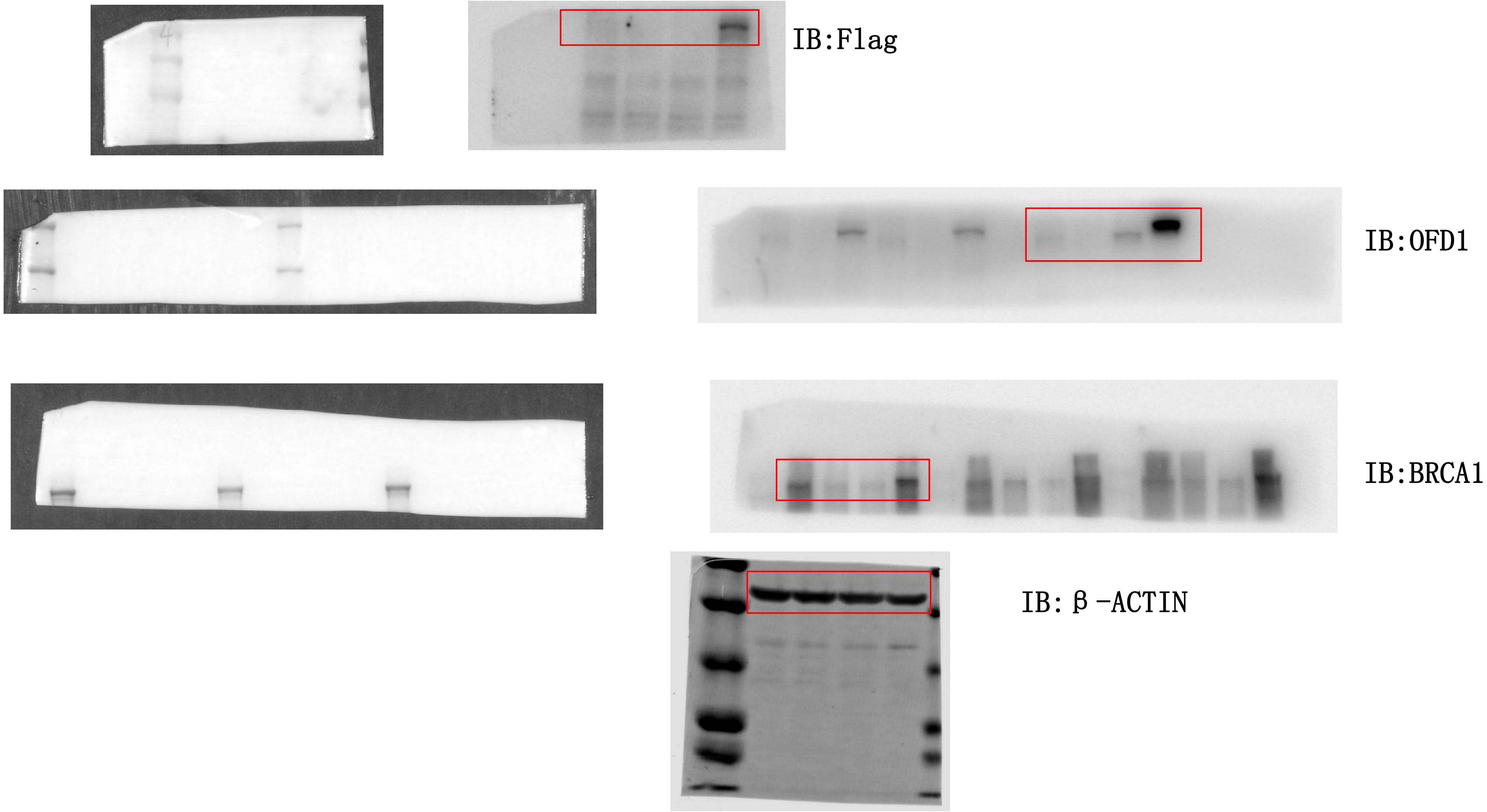

Supplementary Fig 4. i

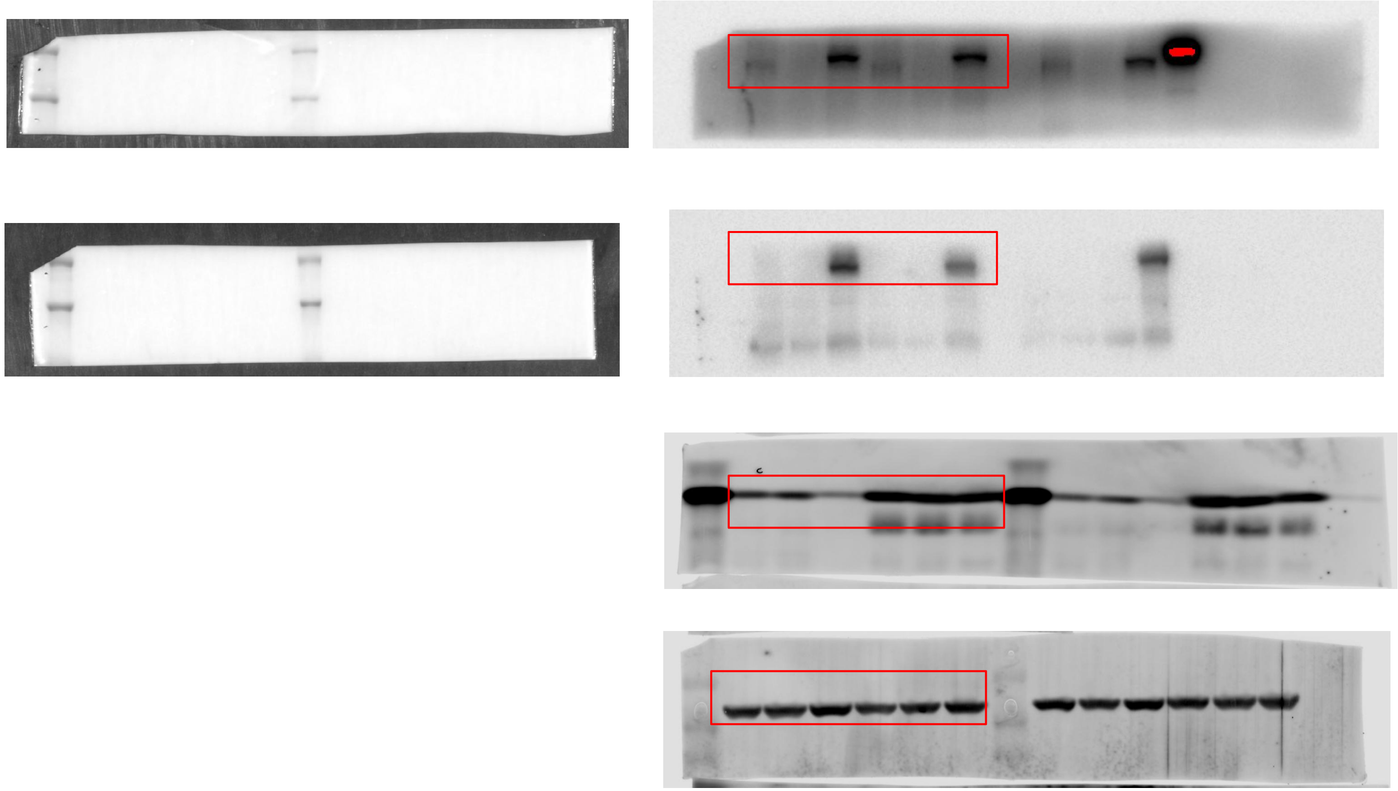

IB:OFD1

IB:Flag

IB: γ H2AX

IB: β-ACTIN

Supplementary Fig 6. d

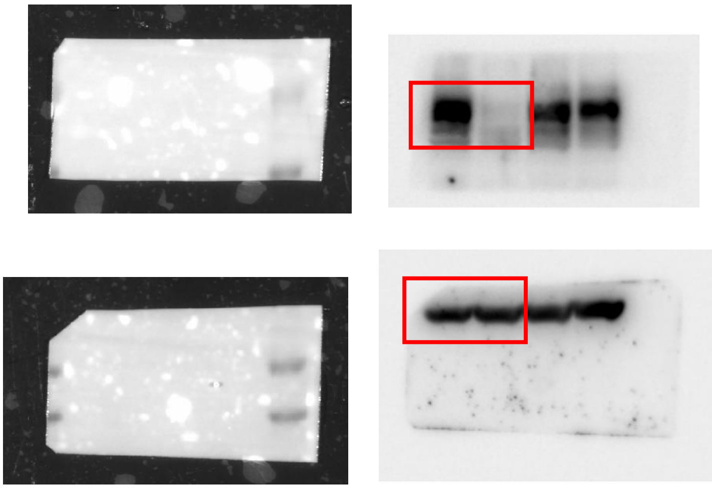

IB: E2F4

IB: β-Actin

Supplementary Fig 6. g

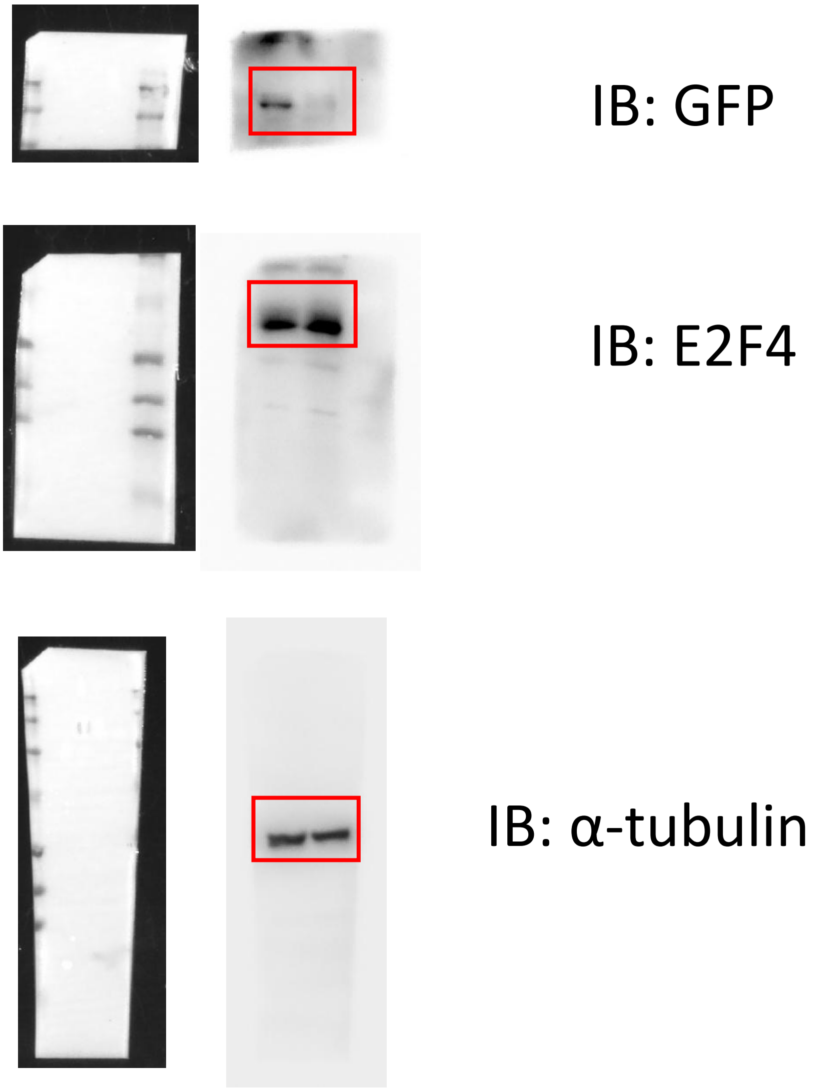

Supplementary Fig 6. i

OFD1-Flag

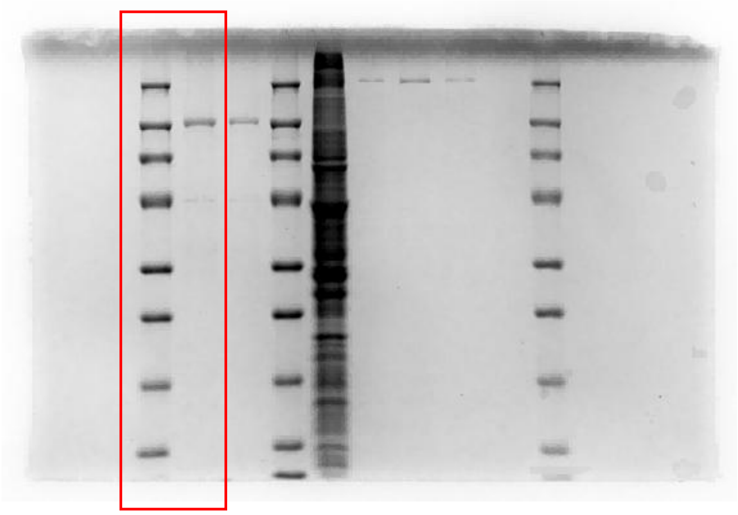

E2F4-Strep-HA

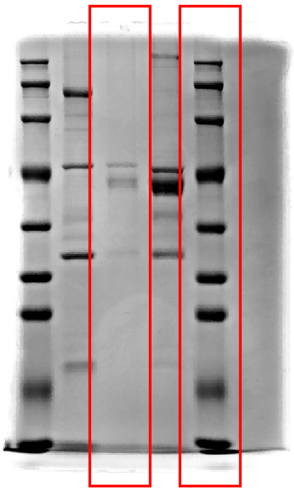

Supplementary Fig 6. j

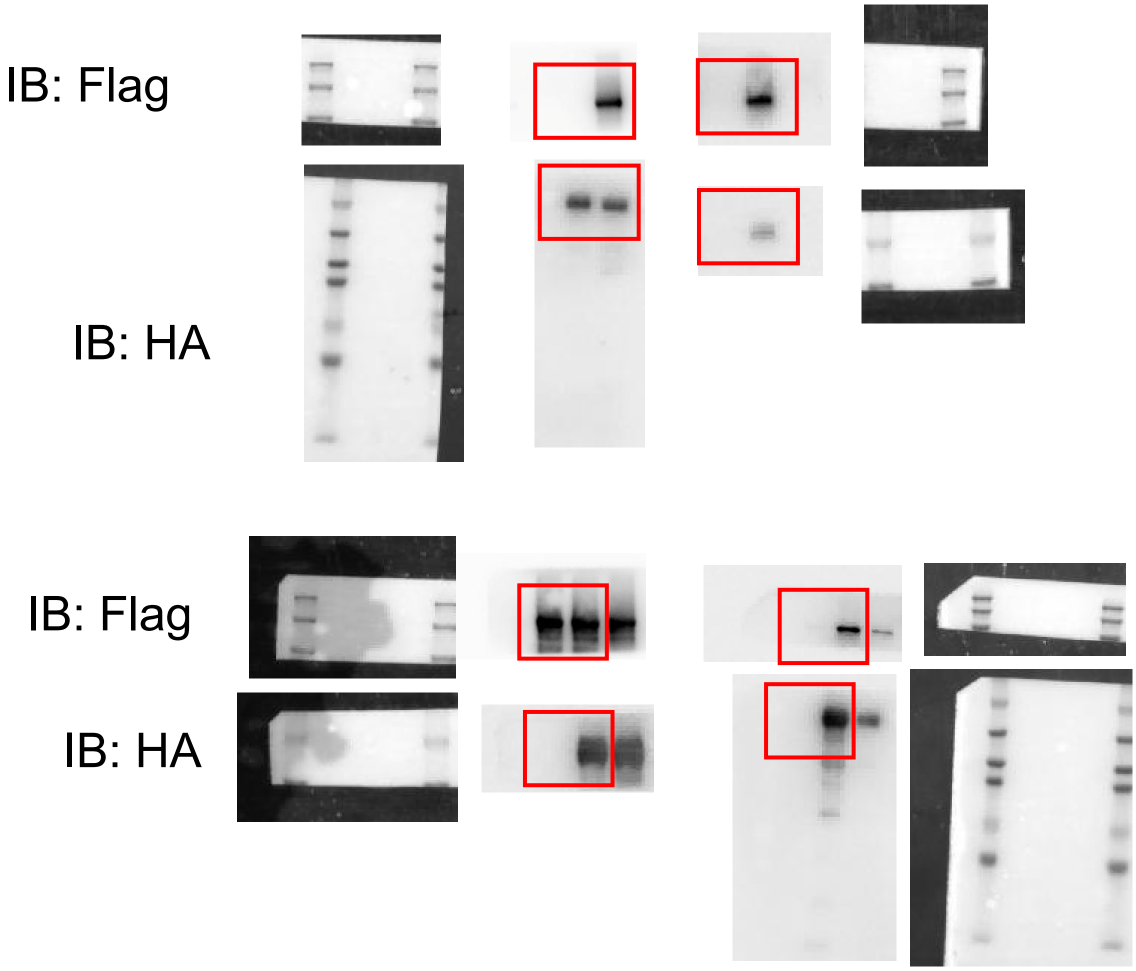

Supplementary Fig 7. b

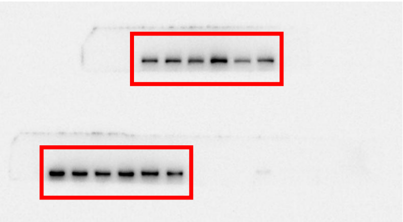

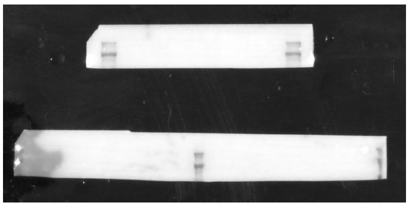

IB: Flag

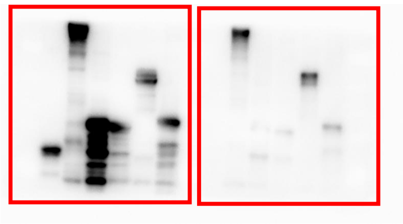

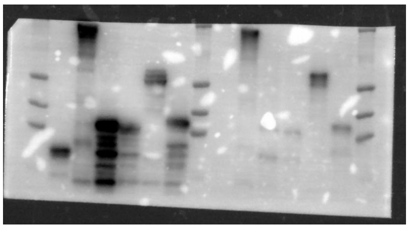

IB: GFP

Supplementary Fig 7. d

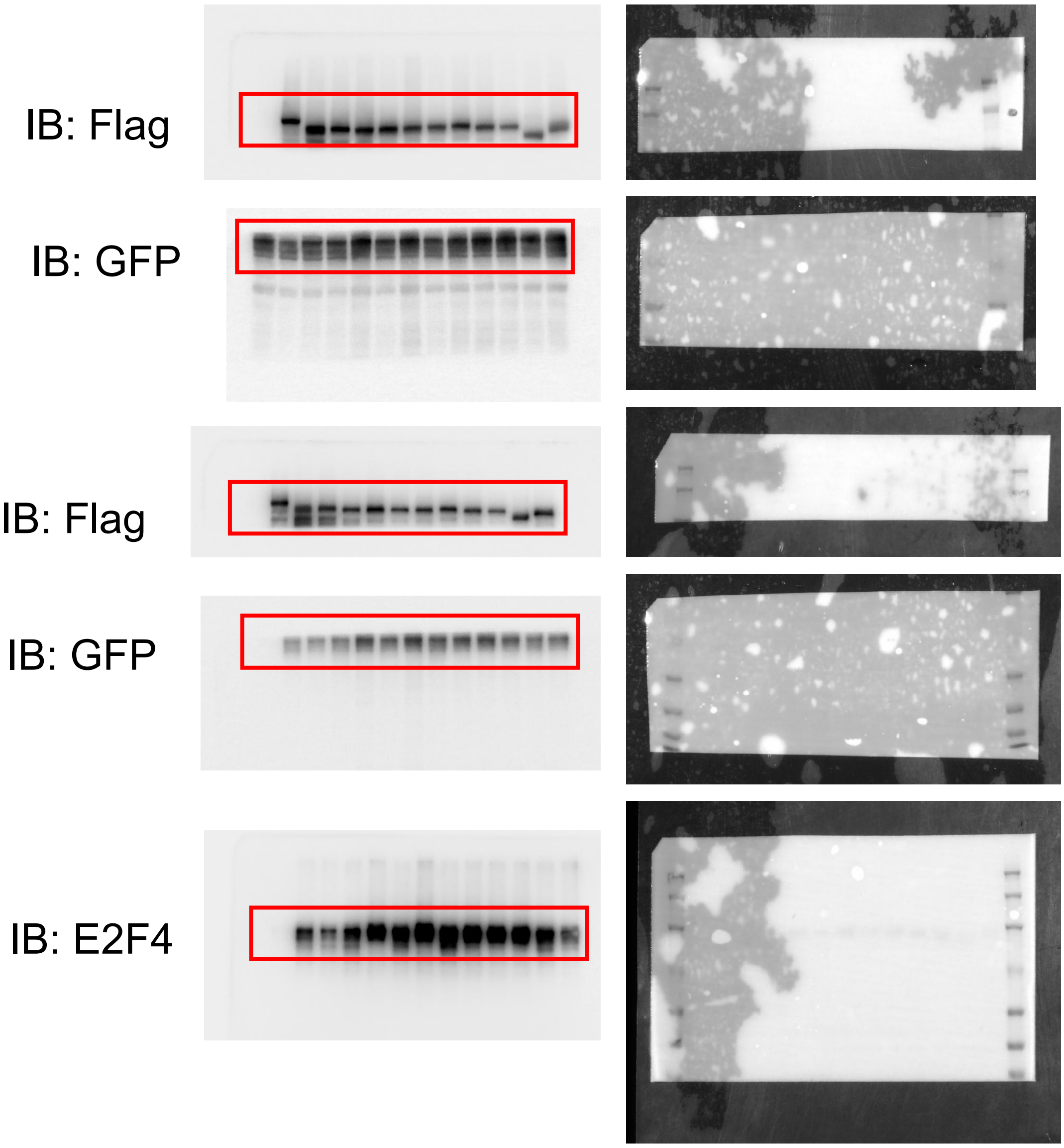

Supplementary Fig 7. e

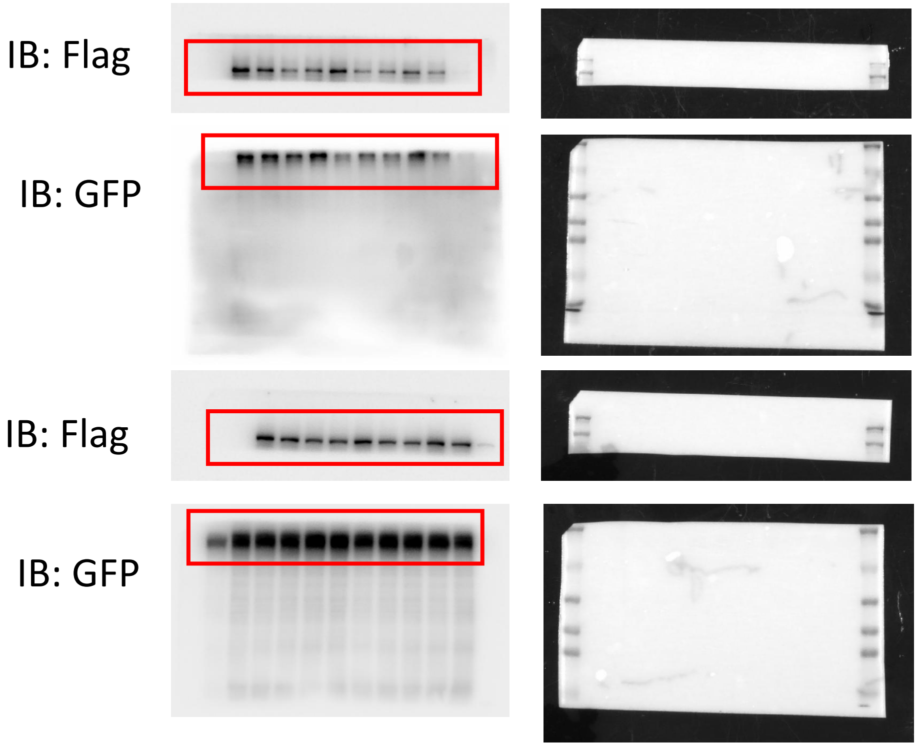

Supplementary Fig 8. a

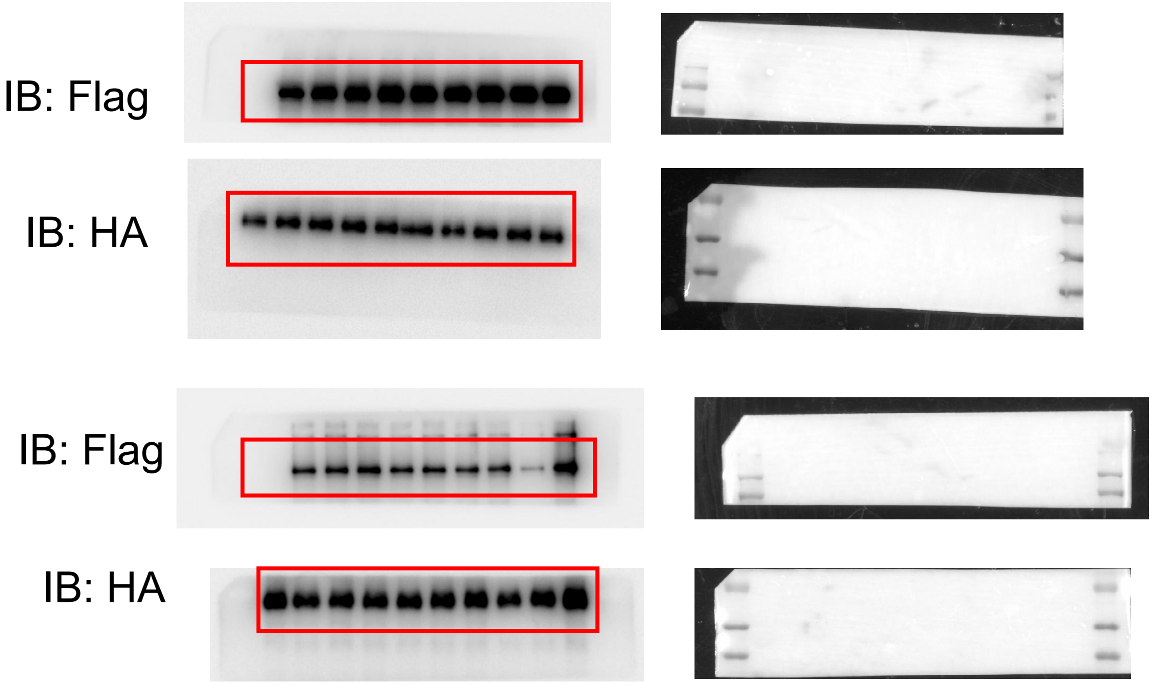

Supplementary Fig 8. b

IB: Flag long exposure

IB: Flag short exposure

IB: HA long exposure

IB: HA short exposure

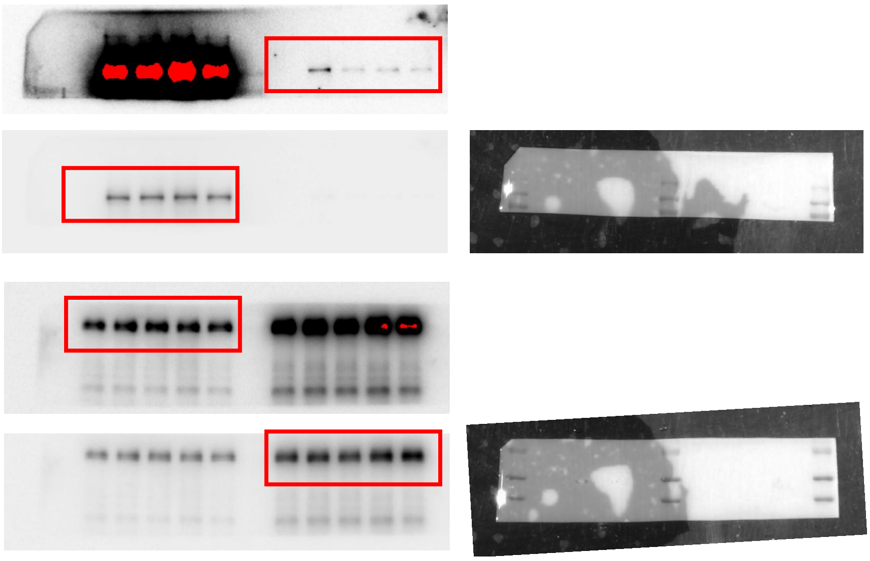

Supplementary Fig 8. c

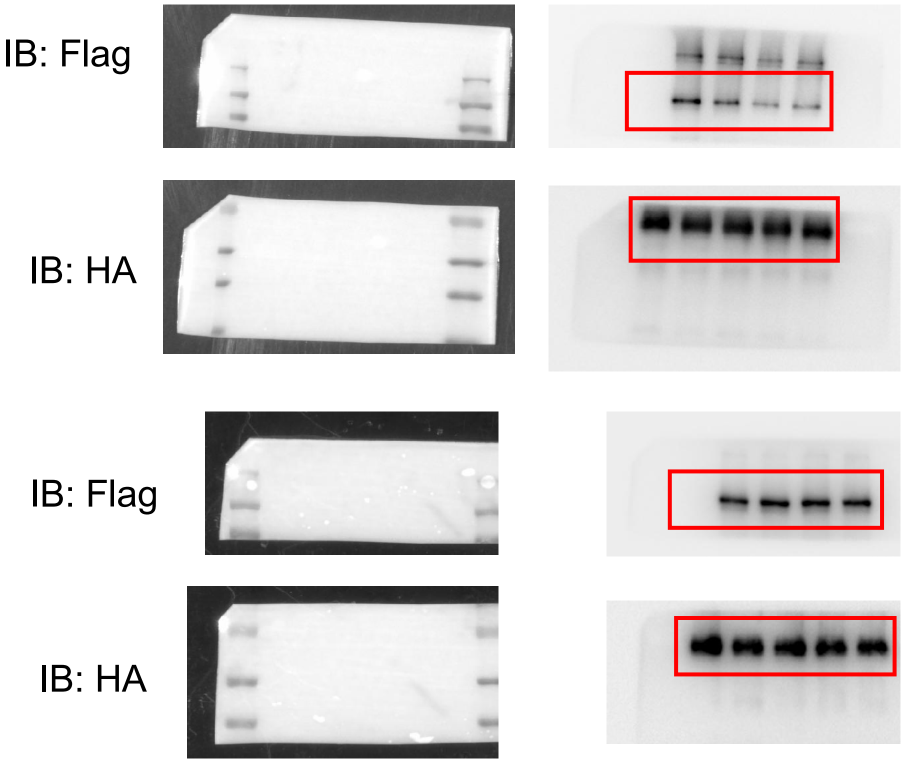

Supplementary Fig 8. g

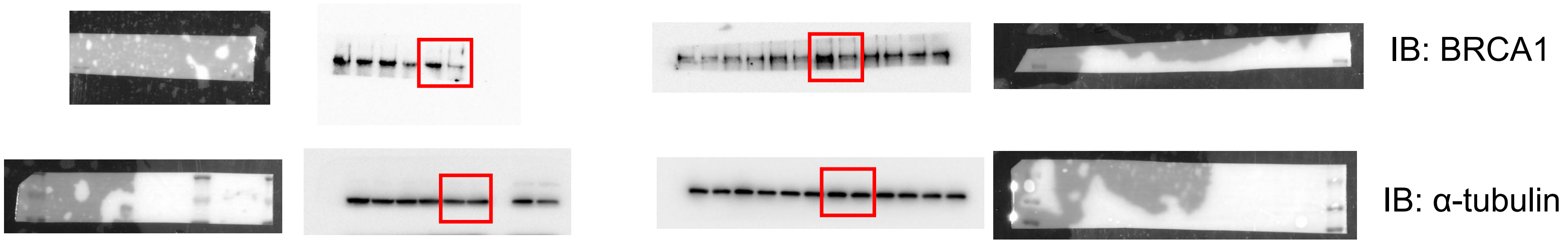

Supplementary Fig 9. b

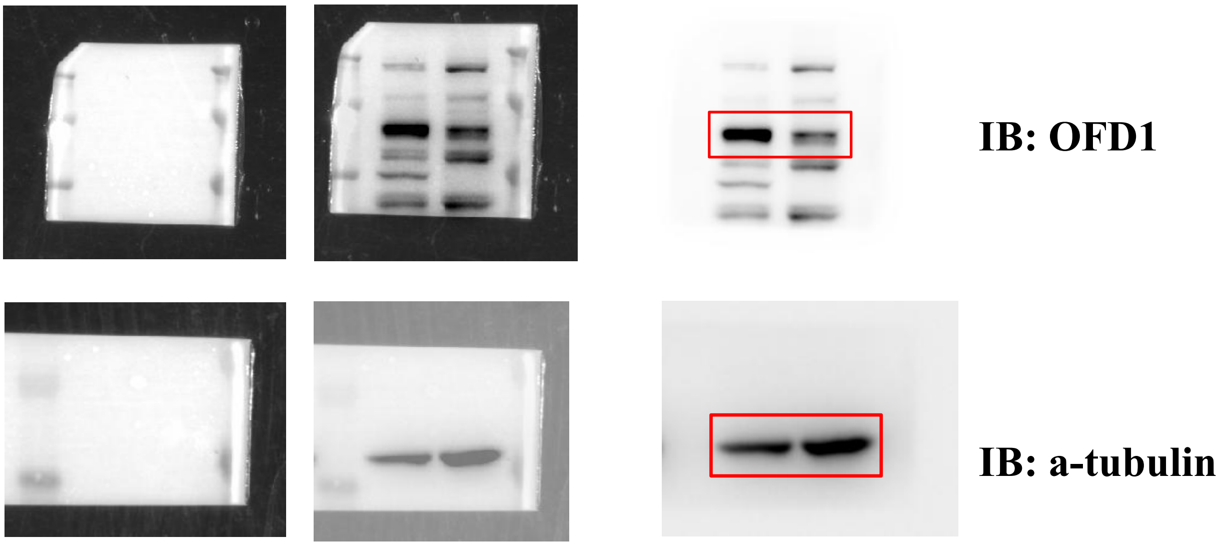

Supplement: Supplementary file 1 — Supplementary Information [file 41467_2025_62295_MOESM1_ESM.pdf]
