# Supplementary material for: OFD1 inhibition induces BRCAness to create a therapeutic vulnerability to PARP inhibition in pancreatic cancer
Source: Nat Commun. 2025 Aug 5;16:7209. doi: 10.1038/s41467-025-62295-8 (PMC12325586; doi:10.1038/s41467-025-62295-8)
Supplement: Supplementary file 2 — Description of Additional Supplementary Files [file 41467_2025_62295_MOESM2_ESM.pdf]

Title: Supplementary Data 1

Description: Comprehensive clinicopathological dataset for pancreatic ductal adenocarcinoma (PDAC) patient's tissue microarray. Data include demographic variables (gender, age), tumor grade, tumoral OFD1 expression status (IHC-classified), overall survival outcomes, and survival duration (months).

Title: Data 2

Description: Synthetic lethality screen data of small molecule inhibitors in inducible OFD1-knockdown MIA PaCa-2 cells (Tet-on shRNA system). Data include inhibitor product names, known targets, IC50 values per group, and IC50 difference% [(MIA - MIA-shOFD1)/MIA ×100%].

Title: Supplementary Data 3

Description: RNA-seq data from three pancreatic cancer cell lines (PANC-1, MIA PaCa-2, PATU8988T) upon OFD1 knockdown. Data include gene names, shOFD1 vs. shControl log2FoldChange, p-values, and adjusted p-values (p.adj).

Title: Supplementary Data 4

Description: qPCR analysis of OFD1 and BRCA1 mRNA expression changes in small-molecule inhibitor-treated MIA PaCa-2 cells. Data include inhibitor names, concentrations, treatment durations, fold-change expression (normalized to control), and OFD1-BRCA1 Pearson correlation coefficients.
