# Supplementary material for: OFD1 inhibition induces BRCAness to create a therapeutic vulnerability to PARP inhibition in pancreatic cancer
Source: Nat Commun. 2025 Aug 5;16:7209. doi: 10.1038/s41467-025-62295-8 (PMC12325586; doi:10.1038/s41467-025-62295-8)
Supplement: Supplementary file 7 — Reporting Summary [file 41467_2025_62295_MOESM7_ESM.pdf]

nature portfolio

Corresponding author(s): Baiyong Shen,Zaiming Tang,Qing Zhong

Last updated by author(s): Jun 5, 2025

Reporting Summary

Nature Portfolio wishes to improve the reproducibility of the work that we publish. This form provides structure for consistency and transparency in reporting. For further information on Nature Portfolio policies, see our Editorial Policies and the Editorial Policy Checklist.

Statistics

For all statistical analyses, confirm that the following items are present in the figure legend, table legend, main text, or Methods section.

n/a	Confirmed	
☐	☒	The exact sample size (*n*) for each experimental group/condition, given as a discrete number and unit of measurement
☐	☒	A statement on whether measurements were taken from distinct samples or whether the same sample was measured repeatedly
☐	☒	The statistical test(s) used AND whether they are one- or two-sided<br>*Only common tests should be described solely by name; describe more complex techniques in the Methods section.*
☒	☐	A description of all covariates tested
☐	☒	A description of any assumptions or corrections, such as tests of normality and adjustment for multiple comparisons
☐	☒	A full description of the statistical parameters including central tendency (e.g. means) or other basic estimates (e.g. regression coefficient) AND variation (e.g. standard deviation) or associated estimates of uncertainty (e.g. confidence intervals)
☐	☒	For null hypothesis testing, the test statistic (e.g. *F*, *t*, *r*) with confidence intervals, effect sizes, degrees of freedom and *P* value noted<br>*Give P values as exact values whenever suitable.*
☒	☐	For Bayesian analysis, information on the choice of priors and Markov chain Monte Carlo settings
☒	☐	For hierarchical and complex designs, identification of the appropriate level for tests and full reporting of outcomes
☐	☒	Estimates of effect sizes (e.g. Cohen's *d*, Pearson's *r*), indicating how they were calculated

Our web collection on statistics for biologists contains articles on many of the points above.

Software and code

Policy information about availability of computer code

Data collection	We used commercial software available with the respective instruments for data collection. Western blot data was collected on an Odyssey machine and Image Studio software (LiCor) or Bio-Rad ChemiDoc Imaging System. Fluorescence images were taken by the FV3000 confocal microscope (Olympus Corporation), or IX83 microscope (Olympus Corporation), or LSM780, or LSM880 laser scanning confocal microscope equipped with an Airyscan module (Zeiss). Tumor burden assessment in the orthotopic model were conducted utilizing the IVIS imaging system. Other data collection methods were specified in the methods section describing each experimental method.
Data analysis	All softwares used to process and plot data in this study were listed in the methods section. Graphs and numerical data (including statistics/ error bars) were analyzed and plotted by Prism (GraphPad, Version 9.1.0); Image J (NIH, Version 1.54k) for the analysis of microscopy data; Immunohistochemistry experiments data was corrected using CaseViewer2.4 and the Image J plug-in IHC Profiler and Toolbox; Flow cytometry data was analyzed using CytExpert(Version2.3.1.22).Comet assay data was analyzed by Image J plug-in OpenComet. Other data analysis methods were specified in the methods section describing each experimental method.

For manuscripts utilizing custom algorithms or software that are central to the research but not yet described in published literature, software must be made available to editors and reviewers. We strongly encourage code deposition in a community repository (e.g. GitHub). See the Nature Portfolio guidelines for submitting code & software for further information.

Data

Policy information about availability of data

All manuscripts must include a data availability statement. This statement should provide the following information, where applicable:

- Accession codes, unique identifiers, or web links for publicly available datasets
- A description of any restrictions on data availability
- For clinical datasets or third party data, please ensure that the statement adheres to our policy

The raw RNA-seq data generated in this study have been deposited in the NCBI Gene Expression Omnibus (GEO) under accession number GSE282746 (https://www.ncbi.nlm.nih.gov/geo/query/acc.cgi?acc=GSE282746). To compare OFD1 expression between pancreatic cancer and normal tissues, we used the following publicly available GEO datasets: GSE15471 (https://www.ncbi.nlm.nih.gov/geo/query/acc.cgi?acc=GSE15471 ; PMID:19260470);GSE71729 (https://www.ncbi.nlm.nih.gov/geo/query/acc.cgi?acc=GSE71729;PMID:26343385);GSE62452 (https://www.ncbi.nlm.nih.gov/geo/query/acc.cgi?acc=GSE62452;PMID:27197190);GSE272362 (https://www.ncbi.nlm.nih.gov/geo/query/acc.cgi?acc=GSE272362;PMID:39294496);GSE71989 (https://www.ncbi.nlm.nih.gov/geo/query/acc.cgi?acc=GSE71989;PMID:27363020);GSE63158 (https://www.ncbi.nlm.nih.gov/geo/query/acc.cgi?acc=GSE63158;PMID:25587357, PMID:28592875). Drug response correlation data with OFD1 mRNA expression were obtained from the Cancer Therapeutics Response Portal (CTRP v2) (https://www.broadinstitute.org/cancer-therapeutics-response-portal). All other data supporting the findings of this study are available within the Article, Supplementary Information, or Source Data files. Source Data are provided with this paper.

Research involving human participants, their data, or biological material

Policy information about studies with human participants or human data. See also policy information about sex, gender (identity/presentation), and sexual orientation and race, ethnicity and racism.

Reporting on sex and gender	Human tissue samples were used in this study, and the sex of each donor was recorded. However, no sex-based analyses were conducted, as the study was not designed or statistically powered to detect sex differences. Information on donor sex is provided in the Supplementary Tables.
Reporting on race, ethnicity, or other socially relevant groupings	The focus of this study was on molecular mechanisms rather than population-level differences; therefore, race and ethnicity were not considered relevant variables for this analysis.
Population characteristics	Human tissue samples were obtained from donors diagnosed with pancreatic ductal adenocarcinoma (PDAC). Donor age, sex, and relevant clinical characteristics were recorded and are summarized in the Supplementary Tables. However, the study was not designed or powered to assess the impact of demographic variables such as age or sex, and no subgroup analyses were performed.
Recruitment	Human pancreatic ductal adenocarcinoma (PDAC) tissue samples were retrospectively collected from patients undergoing surgical resection at the Department of Pancreatic Surgery, Ruijin Hospital, Shanghai Jiao Tong University School of Medicine. All patients provided written informed consent, and sample collection was approved by the institutional ethics committee. Consent documents, including those related to patient-derived xenograft (PDX) models, are provided in the Supplementary Information. Inclusion criteria included histologically confirmed PDA and availability of relevant clinical metadata. No prospective recruitment or randomization was performed.
Ethics oversight	This study complies with all relevant ethical regulations and was approved by the Ethics Committee of Ruijin Hospital and Shanghai Jiao Tong University School of Medicine. All animal study protocols were approved by the Animal Research Committee of Shanghai Jiao Tong University School of Medicine (Reference number: DLAS-MP-ANIM.11). For the patient-derived xenograft study, written informed consent was obtained from all participants, and the study was approved by the Ethics Committee of Ruijin Hospital (Reference number: 2013-70). Pancreatic cancer and normal tissue microarrays (TMA, HPanA180Su03) were prepared by Shanghai Outdo Biotech. The collection and preparation of these tissues were approved by the Scientific Investigation Board of Taizhou Hospital in accordance with the Declaration of Helsinki (Reference number: SHYJS-CP-1901009). Written informed consent was obtained from all human participants prior to sample collection.

Note that full information on the approval of the study protocol must also be provided in the manuscript.

Field-specific reporting

Please select the one below that is the best fit for your research. If you are not sure, read the appropriate sections before making your selection.

[x] Life sciences [ ] Behavioural & social sciences [ ] Ecological, evolutionary & environmental sciences

For a reference copy of the document with all sections, see nature.com/documents/nr-reporting-summary-flat.pdf

Life sciences study design

All studies must disclose on these points even when the disclosure is negative.

Sample size	Sample sizes were determined according to the related references (PMID: 32004442, PMID: 33186520, PMID: 28675157) and chosen based on logistical and experimental experiences. In every case, three or two biologiclal replicates for each experiment were included. All

	experiments were reliably reproduced and the number of samples chosen was sufficient to support meaningful conclusions.
Data exclusions	In general, no data were excluded, except for experimental failures due to control failures and technical problems.
Replication	All microscopic, biochemical, and biological assays were independently repeated at least three times as mentioned in Methods or figure legends. All attempts at replication were successful
Randomization	All groups were randomly assigned,with each group representing a different treatment or condition.
Blinding	Blinding was not applicable to this study as the data grouping and analysis were performed in an unbiased manner. Data was analyzed with common strategies.

Behavioural & social sciences study design

All studies must disclose on these points even when the disclosure is negative.

Study description	*Briefly describe the study type including whether data are quantitative, qualitative, or mixed-methods (e.g. qualitative cross-sectional, quantitative experimental, mixed-methods case study).*
Research sample	*State the research sample (e.g. Harvard university undergraduates, villagers in rural India) and provide relevant demographic information (e.g. age, sex) and indicate whether the sample is representative. Provide a rationale for the study sample chosen. For studies involving existing datasets, please describe the dataset and source.*
Sampling strategy	*Describe the sampling procedure (e.g. random, snowball, stratified, convenience). Describe the statistical methods that were used to predetermine sample size OR if no sample-size calculation was performed, describe how sample sizes were chosen and provide a rationale for why these sample sizes are sufficient. For qualitative data, please indicate whether data saturation was considered, and what criteria were used to decide that no further sampling was needed.*
Data collection	*Provide details about the data collection procedure, including the instruments or devices used to record the data (e.g. pen and paper, computer, eye tracker, video or audio equipment) whether anyone was present besides the participant(s) and the researcher, and whether the researcher was blind to experimental condition and/or the study hypothesis during data collection.*
Timing	*Indicate the start and stop dates of data collection. If there is a gap between collection periods, state the dates for each sample cohort.*
Data exclusions	*If no data were excluded from the analyses, state so OR if data were excluded, provide the exact number of exclusions and the rationale behind them, indicating whether exclusion criteria were pre-established.*
Non-participation	*State how many participants dropped out/declined participation and the reason(s) given OR provide response rate OR state that no participants dropped out/declined participation.*
Randomization	*If participants were not allocated into experimental groups, state so OR describe how participants were allocated to groups, and if allocation was not random, describe how covariates were controlled.*

Ecological, evolutionary & environmental sciences study design

All studies must disclose on these points even when the disclosure is negative.

Study description	*Briefly describe the study. For quantitative data include treatment factors and interactions, design structure (e.g. factorial, nested, hierarchical), nature and number of experimental units and replicates.*
Research sample	*Describe the research sample (e.g. a group of tagged Passer domesticus, all Stenocereus thurberi within Organ Pipe Cactus National Monument), and provide a rationale for the sample choice. When relevant, describe the organism taxa, source, sex, age range and any manipulations. State what population the sample is meant to represent when applicable. For studies involving existing datasets, describe the data and its source.*
Sampling strategy	*Note the sampling procedure. Describe the statistical methods that were used to predetermine sample size OR if no sample-size calculation was performed, describe how sample sizes were chosen and provide a rationale for why these sample sizes are sufficient.*
Data collection	*Describe the data collection procedure, including who recorded the data and how.*
Timing and spatial scale	*Indicate the start and stop dates of data collection, noting the frequency and periodicity of sampling and providing a rationale for these choices. If there is a gap between collection periods, state the dates for each sample cohort. Specify the spatial scale from which the data are taken*
Data exclusions	*If no data were excluded from the analyses, state so OR if data were excluded, describe the exclusions and the rationale behind them, indicating whether exclusion criteria were pre-established.*
Reproducibility	*Describe the measures taken to verify the reproducibility of experimental findings. For each experiment, note whether any attempts to repeat the experiment failed OR state that all attempts to repeat the experiment were successful.*
Randomization	*Describe how samples/organisms/participants were allocated into groups. If allocation was not random, describe how covariates were*

controlled. If this is not relevant to your study, explain why.

Blinding | Describe the extent of blinding used during data acquisition and analysis. If blinding was not possible, describe why OR explain why blinding was not relevant to your study.

Did the study involve field work? ☐ Yes ☐ No

Field work, collection and transport

Field conditions | Describe the study conditions for field work, providing relevant parameters (e.g. temperature, rainfall).

Location | State the location of the sampling or experiment, providing relevant parameters (e.g. latitude and longitude, elevation, water depth).

Access & import/export | Describe the efforts you have made to access habitats and to collect and import/export your samples in a responsible manner and in compliance with local, national and international laws, noting any permits that were obtained (give the name of the issuing authority, the date of issue, and any identifying information).

Disturbance | Describe any disturbance caused by the study and how it was minimized.

Reporting for specific materials, systems and methods

We require information from authors about some types of materials, experimental systems and methods used in many studies. Here, indicate whether each material, system or method listed is relevant to your study. If you are not sure if a list item applies to your research, read the appropriate section before selecting a response.

Materials & experimental systems

n/a	Involved in the study	
☐	☒	Antibodies
☐	☒	Eukaryotic cell lines
☒	☐	Palaeontology and archaeology
☐	☒	Animals and other organisms
☐	☒	Clinical data
☒	☐	Dual use research of concern
☒	☐	Plants

Methods

n/a	Involved in the study	
☒	☐	ChIP-seq
☐	☒	Flow cytometry
☒	☐	MRI-based neuroimaging

Antibodies

Antibodies used

Goat Anti-Mouse Alexa Fluor 488, Jackson ImmunoResearch Laboratories, Cat#115-545-068, 1:500 dilution;
Goat Anti-Rabbit Alexa Fluor 594, Life Technologies, Cat#A-11012, 1:300 dilution;
Goat Anti-Mouse Alexa Fluor 647, Life Technologies, Cat# A32728, 1:300 dilution;
Goat anti-mouse IgG light chain specific HRP, Jackson ImmunoResearch Laboratories, Cat#115-035-174, 1:5000 dilution;
Mouse anti-rabbit IgG light chain specific HRP, Jackson ImmunoResearch Laboratories, Cat#211-032-171, 1:5000 dilution;
Mouse monoclonal anti-α-tubulin, Abcam, Cat#ab7291, 1:10000 dilution;
Mouse monoclonal anti-Flag M2, Sigma Aldrich, Cat#F1804, Clone: M2, monoclonal, 1:3000 dilution (IF), 1:5000 dilution (WB);
Mouse monoclonal GFP tag antibody, Proteintech, Cat #66002-1-Ig,1:10000 dilution (WB);
Mouse monoclonal anti-HA, Sigma Aldrich, Cat#H9658, HA-7, monoclonal, 1:10000 dilution;
Mouse monoclonal β-Actin antibodies, Abcam, Cat# ab 8226,1:10000 dilution (WB);
Mouse polyclonal anti-γH2AX, Cell Signaling Technology, Cat# 80312, 1:300 dilution (IF);
Mouse monoclonal anti-BRCA1, Sata cruz, Cat#sc-6954,1:200 dilution (IHC);
Rabbit polyclonal anti-OFD1, PMID: 33531668, 1:3000 dilution (IF), 1:5000 dilution (WB); 1:3000 dilution (IHC);
Rabbit polyclonal anti-BRCA1, Cell signaling technology, Cat#9010S,1:300 dilution (IF), 1:1000 dilution (WB);
Rabbit polyclonal anti-E2F4, Cell signaling technology, Cat# #40291,1:500 dilution (IF),1:1000(WB);
Rabbit polyclonal anti-RBL2, Cell signaling technology, Cat # 13610, 1:1000 dilution (WB);
Rabbit polyclonal anti-RBBP4, ABclonal, Cat #A3645,1:1000 dilution (WB);
Rabbit polyclonal anti-LIN54, Bethyl Laboratories, Cat #A303-799A, 1:1000 dilution (WB);
Rabbit polyclonal anti-Ofd1(Mouse), Home-made,1:5000 dilution (WB), 1:2000 dilution (IHC);
Rabbit polyclonal anti-KI67 antibody, Proteintech, Cat #27309-1-AP,1:10000 dilution (IHC);
Rabbit polyclonal anti-γH2AX,Cell signaling technology, Cat #60566,1:200 dilution (IF), 1:1000 dilution (WB).

Validation

Rabbit polyclonal anti-OFD1 was validated in literatures (PMID: 24089205, PMID: 36973243); The rabbit polyclonal anti-Ofd1 antibody (specific for mouse) was validated using Western Blot (WB) analysis with Ofd1 knockout cell line KPC1199, as depicted in the manuscript within Extended Data Figure 8b.
All information and validation of commercial antibodies used in this work are available on the manufacturers websites listed below:

Mouse monoclonal anti-α-tubulin, Abcam, Cat#ab7291.
https://www.abcam.cn/products/primary-antibodies/alpha-tubulin-antibody-dm1a-loading-control-ab7291.html

Mouse monoclonal anti-Flag M2, Sigma Aldrich, Cat#F1804, Clone: M2, monoclonal.
https://www.sigmaaldrich.cn/CN/zh/product/sigma/f1804

Mouse monoclonal GFP tag antibody, Proteintech, Cat #66002-1-Ig.
https://www.ptgcn.com/products/eGFP-Antibody-66002-1-Ig.htm

Mouse monoclonal anti-HA, Sigma Aldrich, Cat#H9658, HA-7, monoclonal.
https://www.sigmaaldrich.cn/CN/zh/product/sigma/h9658

Mouse monoclonal β-Actin antibodies, Abcam, Cat# ab 8226
https://www.abcam.cn/products/primary-antibodies/beta-actin-antibody-mabcam-8226-loading-control-ab8226.html

Mouse polyclonal anti-γH2AX, Cell Signaling Technology, Cat# 80312
https://www.cellsignal.cn/products/primary-antibodies/phospho-histone-h2a-x-ser139-d7t2v-mouse-mab/80312

Mouse monoclonal anti-BRCA1, Sata cruz, Cat#sc-6954
https://www.scbt.com/zh/p/brca1-antibody-d-9
Rabbit polyclonal anti-BRCA1, Cell signaling technology, Cat#9010S
https://www.cellsignal.cn/products/primary-antibodies/brca1-antibody/9010

Rabbit polyclonal anti-E2F4, Cell signaling technology, Cat#40291
https://www.cellsignal.cn/products/primary-antibodies/e2f4-e3g2g-rabbit-mab/40291

Rabbit polyclonal anti-RBL2, Cell signaling technology, Cat # 13610
https://www.cellsignal.cn/products/primary-antibodies/rbl2-d9t7m-rabbit-mab/13610

Rabbit polyclonal anti-RBBP4, ABclonal, Cat #A3645
https://abclonal.com.cn/catalog/A3645

Rabbit polyclonal anti-LIN54, Bethyl Laboratories, Cat #A303-799A
https://www.biomol.com/products/antibodies/primary-antibodies/general/anti-lin54-a303-799a-t?fs=1127707153

Rabbit polyclonal anti-KI67 antibody, Proteintech, Cat #27309-1-AP
https://www.ptgcn.com/products/KI67-Antibody-27309-1-AP.htm

Rabbit polyclonal anti-γH2AX, Cell signaling technology, Cat #60566,1:200 dilution (IF), 1:1000 dilution (WB).
https://www.cellsignal.cn/products/primary-antibodies/phospho-histone-h2a-x-ser139-20e3-rabbit-mab/9718

Eukaryotic cell lines

Policy information about cell lines and Sex and Gender in Research

Cell line source(s): The cell lines MIA PaCa-2, BxPC-3, HPAC, SW1990, PANC1, PATU8988T, PANC1005, MDA-MB231, PC3, A2780, HPAFII, and HEK293 were procured from the American Type Culture Collection (ATCC). Additionally, the KPC1199 cell line, a derivative of the KPC model of pancreatic ductal adenocarcinoma (PDAC), was obtained from Hingorani et al., as reported in Cancer Cell in 2005 (PMID: 15894267).

Authentication: The cell lines exhibited morphological features, growth characteristics and phenotypic responses consistent with original description of these cell lines. All cell lines were authenticated by ATCC STR testing.

Mycoplasma contamination: All cell lines used in experiments were tested negative for mycoplasma contamination.

Commonly misidentified lines (See ICLAC register): No commonly misidentified cell lines were used in our study.

Palaeontology and Archaeology

Specimen provenance: *Provide provenance information for specimens and describe permits that were obtained for the work (including the name of the issuing authority, the date of issue, and any identifying information). Permits should encompass collection and, where applicable, export.*

Specimen deposition: *Indicate where the specimens have been deposited to permit free access by other researchers.*

Dating methods: *If new dates are provided, describe how they were obtained (e.g. collection, storage, sample pretreatment and measurement), where*

they were obtained (i.e. lab name), the calibration program and the protocol for quality assurance OR state that no new dates are provided.

☐ Tick this box to confirm that the raw and calibrated dates are available in the paper or in Supplementary Information.

Ethics oversight	This study complies with all relevant ethical regulations and was approved by the Ethics Committee of Ruijin Hospital and Shanghai Jiao Tong University School of Medicine. Reference number for PDX samples: 2013-70; reference number for PDAC and normal tissue array: SHYJS-CP-1901009; policy number for animal models: DLAS-MP-ANIM.11. All participants provided informed consent. Humane endpoint was determined for animal models according to the maximun tumor burden, body weight loss, action abnormality, ascites, etc.

Note that full information on the approval of the study protocol must also be provided in the manuscript.

Animals and other research organisms

Policy information about studies involving animals; ARRIVE guidelines recommended for reporting animal research, and Sex and Gender in Research

Laboratory animals	6- to 8-week-old female and male BALB/c-nu mice were used in this study for Xenografts, Orthotopic tumor models and PDX (Patient-derived xenografts). The Pdx1-Cre, LSL-KrasG12D/+, and LSL-Trp53R172H/＋ genetically engineered mice were obtained from The Jackson Laboratory (Bar Harbor, ME). The Ofd1(flox/flox) mice were generated using ES gene-targeting technology, and subsequently hybridized with the KPC mouse model. All mice were kept in group housing (3-5 mice per cage) in a specific pathogen-free facility with controlled environmental conditions of humidity (50±10%), lighting (a 12-h light/dark cycle) and controlled temperature (21±1° C) at the animal facility, Shanghai Jiaotong University School of Medicine.
Wild animals	No wild animals were used in the study.
Reporting on sex	The female and male BALB/c-nu mice were used in this study; The female and male Pdx1-Cre, LSL-KrasG12D/+, Trp53R172H/＋and Ofd1(flox/flox) mice were used in this study.
Field-collected samples	No field collected samples were used in the study.
Ethics oversight	All of the mouse experiments were performed according to the guidelines of the Institutional Animal Care and Use Committee (IACUC) at Shanghai Jiao Tong University School of Medicine.

Note that full information on the approval of the study protocol must also be provided in the manuscript.

Clinical data

Policy information about clinical studies

All manuscripts should comply with the ICMJE guidelines for publication of clinical research and a completed CONSORT checklist must be included with all submissions.

Clinical trial registration	The tissue arrays containing tissues from 90 paired pancreatic cancer and normal tissue samples (HPanA180Su03) were used to examine the expression profiles of OFD1 and BRCA1 by immunohistochemistry (IHC). Human pancreatic ductal adenocarcinoma (PDAC) tissue samples for this PDX model study were sourced from two patients who underwent surgical resections at Ruijin Hospital, affiliated with Shanghai Jiao Tong University School of Medicine in Shanghai, China. These samples were subsequently implanted into nude mice to generate the PDX models.
Study protocol	For PDAC microarray IHC analysis, Pancreatic cancer with corresponding normal tissue microarray (TMA) sections were prepared by Shanghai Outdo Biotech Co. Ltd. (Shanghai, China).<br>For PDX study, human pancreatic ductal adenocarcinoma (PDAC) tissue samples were sourced from two patients who underwent surgical resections at Ruijin Hospital, affiliated with Shanghai Jiao Tong University School of Medicine in Shanghai, China. Pancreatic tumors were collected in DMEM medium (without FBS) and kept on wet ice for engraftment within 24 hours after resection. Approximately 1 mm3 tumor tissue was implanted subcutaneously into the flank region of athymic BALB/c nude mice using a trocar. A portion of each tumor was used to for BRCA1/2 exon sequencing. For tumor subculture, when the tumor volume reached 600 mm3, mice were anesthetized, and tumor tissue was collected for serial transplantation to the next generation.<br>For PDAC microarray IHC analysis, Pancreatic cancer with corresponding normal tissue microarray (TMA) sections were prepared by Shanghai Outdo Biotech Co. Ltd. (Shanghai, China).
Data collection	Pancreatic cancer and normal tissue microarrays (TMA, HPanA180Su03) were prepared by Shanghai Outdo Biotech. The collection and preparation of these tissues were approved by the Scientific Investigation Board of Taizhou Hospital in accordance with the Declaration of Helsinki (Reference number: SHYJS-CP-1901009). Written informed consent was obtained from all human participants prior to sample collection.<br>The human PDAC patient-derived xenograft (PDX) tissue samples utilized in this study were sourced from patients who underwent surgical resections at Ruijin Hospital, which is affiliated with Shanghai Jiao Tong University School of Medicine in Shanghai, China. Furthermore, the PDX model animal experiments were conducted at the animal facility of Shanghai Jiao Tong University School of Medicine. The entire study was reviewed and approved by the Ethics Committee of Ruijin Hospital and Shanghai Jiao Tong University School of Medicine（Reference number:2013-70）.
Outcomes	For PDAC microarray IHC staining were scored by two independent pathologists who were blinded to the clinical characteristics of the patients. using ImageJ (NIH) IHC scoring system was based on the intensity and extent of staining, as a 3-tier scale (0; negative to weak, 1; moderate, 2; strong). For OFD1 expression and survival analysis, Strong staining was defined as “High”, and the rest of the

staining was defined as "Low".
For PDX studies, PDX tumors (~100 mm3) were treated with Olaparib (60mg/kg) or placebo three times a week, in combination with AAV-shOFD1 or AAV-shCtr treatment through intratumoral injection. Tumor size and body weight were evaluated twice a week. At the end of the experiment, all tumors were harvested and weighed. Tumor volumes were calculated using the formula: Tumor volume (mm3) = 0.52 × (Length × Width2).

Dual use research of concern

Policy information about dual use research of concern

Hazards

Could the accidental, deliberate or reckless misuse of agents or technologies generated in the work, or the application of information presented in the manuscript, pose a threat to:

No	Yes	
☐	☐	Public health
☐	☐	National security
☐	☐	Crops and/or livestock
☐	☐	Ecosystems
☐	☐	Any other significant area

Experiments of concern

Does the work involve any of these experiments of concern:

No	Yes	
☐	☐	Demonstrate how to render a vaccine ineffective
☐	☐	Confer resistance to therapeutically useful antibiotics or antiviral agents
☐	☐	Enhance the virulence of a pathogen or render a nonpathogen virulent
☐	☐	Increase transmissibility of a pathogen
☐	☐	Alter the host range of a pathogen
☐	☐	Enable evasion of diagnostic/detection modalities
☐	☐	Enable the weaponization of a biological agent or toxin
☐	☐	Any other potentially harmful combination of experiments and agents

Plants

Seed stocks	N/A
Novel plant genotypes	N/A
Authentication	N/A

ChIP-seq

Data deposition

☐ Confirm that both raw and final processed data have been deposited in a public database such as GEO.

☐ Confirm that you have deposited or provided access to graph files (e.g. BED files) for the called peaks.

Data access links<br>*May remain private before publication.*	*For "Initial submission" or "Revised version" documents, provide reviewer access links. For your "Final submission" document, provide a link to the deposited data.*
Files in database submission	*Provide a list of all files available in the database submission.*

Genome browser session
(e.g. UCSC)

Provide a link to an anonymized genome browser session for "Initial submission" and "Revised version" documents only, to enable peer review. Write "no longer applicable" for "Final submission" documents.

Methodology

Replicates

Describe the experimental replicates, specifying number, type and replicate agreement.

Sequencing depth

Describe the sequencing depth for each experiment, providing the total number of reads, uniquely mapped reads, length of reads and whether they were paired- or single-end.

Antibodies

Describe the antibodies used for the ChIP-seq experiments; as applicable, provide supplier name, catalog number, clone name, and lot number.

Peak calling parameters

Specify the command line program and parameters used for read mapping and peak calling, including the ChIP, control and index files used.

Data quality

Describe the methods used to ensure data quality in full detail, including how many peaks are at FDR 5% and above 5-fold enrichment.

Software

Describe the software used to collect and analyze the ChIP-seq data. For custom code that has been deposited into a community repository, provide accession details.

Flow Cytometry

Plots

Confirm that:

☐ The axis labels state the marker and fluorochrome used (e.g. CD4-FITC).

☒ The axis scales are clearly visible. Include numbers along axes only for bottom left plot of group (a 'group' is an analysis of identical markers).

☒ All plots are contour plots with outliers or pseudocolor plots.

☒ A numerical value for number of cells or percentage (with statistics) is provided.

Methodology

Sample preparation

pCVL Traffic Light Reporter was packaged into lentivirus and transduced into pancreatic cancer cell line MIA PaCa-2. Stable cell lines were selected by puromycin for 1 week, followed by transfected with I-SceI expression plasmid. After treatment, cells were harvested and washed with chilled PBS and analyzed by flow cytometry (FACS) .

Instrument

BD FACSCalibur

Software

CytExpert(Version2.3.1.22)

Cell population abundance

At least 50,000 cells were counted.

Gating strategy

FSC-A and FSC-H was used to identify single cells, FITC (emission wavelength: 495 nm) is utilized for labeling GFP-labeled cells (HR events), while ECD (emission wavelength: 565 nm) is employed for labeling mCherry-labeled cells (NHEJ events).

☒ Tick this box to confirm that a figure exemplifying the gating strategy is provided in the Supplementary Information.

Magnetic resonance imaging

Experimental design

Design type

Indicate task or resting state; event-related or block design.

Design specifications

Specify the number of blocks, trials or experimental units per session and/or subject, and specify the length of each trial or block (if trials are blocked) and interval between trials.

Behavioral performance measures

State number and/or type of variables recorded (e.g. correct button press, response time) and what statistics were used to establish that the subjects were performing the task as expected (e.g. mean, range, and/or standard deviation across subjects).

Acquisition

Imaging type(s) | *Specify: functional, structural, diffusion, perfusion.*

Field strength | *Specify in Tesla*

Sequence & imaging parameters | *Specify the pulse sequence type (gradient echo, spin echo, etc.), imaging type (EPI, spiral, etc.), field of view, matrix size, slice thickness, orientation and TE/TR/flip angle.*

Area of acquisition | *State whether a whole brain scan was used OR define the area of acquisition, describing how the region was determined.*

Diffusion MRI ☐ Used ☐ Not used

Preprocessing

Preprocessing software | *Provide detail on software version and revision number and on specific parameters (model/functions, brain extraction, segmentation, smoothing kernel size, etc.).*

Normalization | *If data were normalized/standardized, describe the approach(es): specify linear or non-linear and define image types used for transformation OR indicate that data were not normalized and explain rationale for lack of normalization.*

Normalization template | *Describe the template used for normalization/transformation, specifying subject space or group standardized space (e.g. original Talairach, MNI305, ICBM152) OR indicate that the data were not normalized.*

Noise and artifact removal | *Describe your procedure(s) for artifact and structured noise removal, specifying motion parameters, tissue signals and physiological signals (heart rate, respiration).*

Volume censoring | *Define your software and/or method and criteria for volume censoring, and state the extent of such censoring.*

Statistical modeling & inference

Model type and settings | *Specify type (mass univariate, multivariate, RSA, predictive, etc.) and describe essential details of the model at the first and second levels (e.g. fixed, random or mixed effects; drift or auto-correlation).*

Effect(s) tested | *Define precise effect in terms of the task or stimulus conditions instead of psychological concepts and indicate whether ANOVA or factorial designs were used.*

Specify type of analysis: ☐ Whole brain ☐ ROI-based ☐ Both

Statistic type for inference | *Specify voxel-wise or cluster-wise and report all relevant parameters for cluster-wise methods.*

(See Eklund et al. 2016)

Correction | *Describe the type of correction and how it is obtained for multiple comparisons (e.g. FWE, FDR, permutation or Monte Carlo).*

Models & analysis

n/a	Involved in the study
☐	☐ Functional and/or effective connectivity
☐	☐ Graph analysis
☐	☐ Multivariate modeling or predictive analysis

Functional and/or effective connectivity | *Report the measures of dependence used and the model details (e.g. Pearson correlation, partial correlation, mutual information).*

Graph analysis | *Report the dependent variable and connectivity measure, specifying weighted graph or binarized graph, subject- or group-level, and the global and/or node summaries used (e.g. clustering coefficient, efficiency, etc.).*

Multivariate modeling and predictive analysis | *Specify independent variables, features extraction and dimension reduction, model, training and evaluation metrics.*
